# Supplementary material for: Incidental findings on brain imaging and blood tests: results from the first phase of Insight 46, a prospective observational substudy of the 1946 British birth cohort
Source: BMJ Open. 2019 Jul 31;9(7):e029502. doi: 10.1136/bmjopen-2019-029502 (PMC6678011; doi:10.1136/bmjopen-2019-029502)
Supplement: Supplementary data [file bmjopen-2019-029502supp001.pdf]

Supplementary File 1. Life course data required for Insight 46 eligibility*

1. Attendance at a clinic visit at age 60-64.
2. Parental socioeconomic position: at least one indicator of occupational social class or education.
3. Cognition: memory and processing speed from the 60-64 clinic visit AND at least one set of measures at ages 8, 11 or 15.
4. Early physical growth trajectories: birth weight and at least one measure of height and weight at ages 4-15
5. Educational attainment: highest qualification by age 26.
6. Mental health: teacher ratings of behaviour and temperament at ages 13 or 15, and at least one measure of affective symptoms at ages 36, 43, 53 or 60-64.
7. Blood pressure, lung function, adult height and weight: at least one measure of each at ages 36, 43, 53 or 60-64.
8. Health behaviours: at least one measure of smoking and physical exercise at ages 36, 43, 53 or 60-64.
9. Blood: either age 53 or 60-64 samples.

*Criteria were relaxed towards end of study to allow recruitment of 62 participants without a measure of lung function, smoking or physical exercise, in order to achieve recruitment target
