# Supplementary material for: Incidental findings on brain imaging and blood tests: results from the first phase of Insight 46, a prospective observational substudy of the 1946 British birth cohort
Source: BMJ Open. 2019 Jul 31;9(7):e029502. doi: 10.1136/bmjopen-2019-029502 (PMC6678011; doi:10.1136/bmjopen-2019-029502)
Supplement: Supplementary data [file bmjopen-2019-029502supp002.pdf]

MRC Unit for Lifelong Health and Ageing

UCL

# MRC National Survey of Health and Development Neuroimaging sub-study

## Participant Consent Form

Version 2.3:17-January-2015

***PLEASE INITIAL BOXES – where you agree***

1. I confirm that I have read and understand the information booklet for this follow-up of the MRC National Survey of Health and Development (version 2.3 dated 17 January 2015) I have had the opportunity to consider the information, ask questions about the study and have had these answered satisfactorily. ☐
2. I understand that certain assessments will be video recorded. ☐
3. I understand that relevant sections of any of my medical notes and data collected during the study may be looked at by responsible individuals from the MRC National Survey of Health and Development, from regulatory authorities or from the NHS Trust where it is relevant to my taking part in this research. I give permission for these individuals to have access to my records. ☐
4. I agree to have my research data electronically linked to other databases including those from primary care, Hospital Episode Statistics, NHS Health and Social Care Information Centre, research, clinical and administrative registries. To do this, I understand that my name, postcode, date of birth and NHS number will be shared with the NHS Health and Social Care Information Centre. ☐
5. I understand that the blood and urine samples will be used for research purposes only, and that no information found in the DNA will be given to me. ☐
6. I agree to the information and samples being used for health research carried out by the study team, and by scientists with research projects approved by the study's Data Sharing Committee. I understand that all analysis will be carried out and published using data only in numeric and anonymised form. ☐
7. I agree to the information and samples being held by UCL as the Data Controller. UCL is responsible for ensuring all data are securely stored, handled and used in accordance with the Data Protection Act. ☐
8. I agree that the results of the tests and measures listed in the GP letter be sent to my GP. ☐
9. I agree to take part in the above study. ☐

| Name of study member | Date | Signature |
|---|---|---|
| Name of person taking consent | Date | Signature |

**Thank you for agreeing to participate in the MRC NSHD Neuroimaging sub-study**

When completed, 1 for study member, 1 for Institute of Neurology, and original for MRC NSHD

MRC Unit for Lifelong Health and Ageing at UCL 33 Bedford Place London WC1B 5JU
Telephone: 020 7670 5700 www.nshd.mrc.ac.uk

MRC Unit for Lifelong Health and Ageing

UCL

# MRC National Survey of Health and Development Neuroimaging sub-study

## PET/MRI Consent Form

Version 1.2:17-January-2015

***PLEASE INITIAL BOXES – where you agree***

1. I confirm that I have read and understood the participant information sheet (version 2.3 dated 17 January 2015) for the above study and have had the opportunity to ask questions. ☐
2. I understand that my participation is voluntary and that I am free to withdraw at any time, without giving any reason, without my medical care or legal right being affected. ☐
3. I agree to my GP being informed of my participation in the study and that my GP may be informed of any unexpected findings on the research scan. ☐
4. I understand that unexpected findings on the MR scan may be discussed with me. ☐
5. I agree to take part in the study. ☐

| ______________________ | __________ | ______________________ |
|---|---|---|
| Name of study member | Date | Signature |
| ______________________ | __________ | ______________________ |
| Name of person taking consent | Date | Signature |

**Thank you for agreeing to participate in the MRC NSHD Neuroimaging sub-study**

When completed, 1 for study member, 1 for Institute of Neurology, and original for MRC NSHD

MRC Unit for Lifelong Health and Ageing at UCL 33 Bedford Place London WC1B 5JU
Telephone: 020 7670 5700 www.nshd.mrc.ac.uk
