# Supplementary material for: Incidental findings on brain imaging and blood tests: results from the first phase of Insight 46, a prospective observational substudy of the 1946 British birth cohort
Source: BMJ Open. 2019 Jul 31;9(7):e029502. doi: 10.1136/bmjopen-2019-029502 (PMC6678011; doi:10.1136/bmjopen-2019-029502)
Supplement: Supplementary data [file bmjopen-2019-029502supp003.pdf]

Acute brain infarction	◯ No ◯ Yes
Acute brain haemorrhage	◯ No ◯ Yes
Intracranial mass lesion	◯ No ◯ Yes
Suspected intracranial aneurysm or vascular malformation (inc. cavernomata	◯ No ◯ Yes
Colloid cyst of the 3rd ventricle	◯ No ◯ Yes
Acute hydrocephalus	◯ No ◯ Yes
Significant sinus disease with suspicion of underlying pathology (e.g. unilateral sinus opacification	◯ No ◯ Yes
Other unexpected, serious, or life-threatening findings	◯ No ◯ Yes
Additional comments	
Flagged For Review	No

Save Revert Cancel
