# Supplementary material for: Incidental findings on brain imaging and blood tests: results from the first phase of Insight 46, a prospective observational substudy of the 1946 British birth cohort
Source: BMJ Open. 2019 Jul 31;9(7):e029502. doi: 10.1136/bmjopen-2019-029502 (PMC6678011; doi:10.1136/bmjopen-2019-029502)
Supplement: Supplementary data [file bmjopen-2019-029502supp004.pdf]

Supplementary File 4. Findings flagged by radiologists that were not considered reportable

- Moderately severe small vessel disease
- Extensive cortical and subcortical chronic changes, right cerebral lateral convexity – likely sequelae of chronic embolic infarcts in right M4 territories
- Radiological features (ventricular enlargement, sulcal crowding at the vertex, widening of the sylvian fissures) which could indicate normal pressure hydrocephalus in the appropriate clinical context
- Several T2-hyperintense lesions in the supratentorial white matter, including juxtacortical and periventricular foci, with a further lesion in the upper cervical spinal cord, suggestive of an inflammatory-demyelinating process
- Generalised frontoparietal volume loss with the impression of more marked midbrain atrophy, which in the appropriate clinical context raises the possibility of a neurodegenerative process
- Generalised neuroparenchymal volume loss, slightly more marked in the parietal lobes
- Altered FLAIR signal within some of the sulcal spaces of the left frontal lobe, which raises the possibility of previous subarachnoid blood or a leptomeningeal process
- Generalised involutional change (featuring areas of isolated sulcal widening) with more marked hippocampal volume loss
- T2-hyperintense intra-diploic lesion within the left frontal bone without associated cortical destruction or intracranial/extracalvarial extension, likely represents a haemangioma
- Moderately severe small vessel disease
- Relatively extensive patch of T2-hyperintense signal change in the cerebral white matter and pons, along with a cribiform appearance of the basal ganglia, in keeping with moderately severe small vessel disease
- Mild background small vessel disease, with a more confluent focus within the white matter deep to the right peri-rolandic region, which likely represents a focus of established subcortical ischaemia
- An ossified right temporal dural-based mass, likely meningioma
- Disproportionate neuroparenchymal volume loss affecting the left cerebral hemisphere, particularly the perisylvian region and temporal lobe, suspicious for neurodegeneration
- Moderate demyelination. Mild disproportionate cortical atrophy.
