# Supplementary material for: Incidental findings on brain imaging and blood tests: results from the first phase of Insight 46, a prospective observational substudy of the 1946 British birth cohort
Source: BMJ Open. 2019 Jul 31;9(7):e029502. doi: 10.1136/bmjopen-2019-029502 (PMC6678011; doi:10.1136/bmjopen-2019-029502)
Supplement: Supplementary data [file bmjopen-2019-029502supp005.pdf]

Supplementary File 5. Data regarding follow-up and subsequent outcomes of incidental findings on brain MRI

**Incidental Finding**	**Outcome**	**Source**
Possible aneurysm right ICA	Known about previously and already being followed up locally.	Participant
Probable right MCA bifurcation aneurysm	Seen by neurosurgery. Did not have further imaging or follow-up.	Participant
Possible right PCOMA aneurysm	Seen by neurosurgery. Had angiogram which showed 8mm right ICA aneurysm. Subsequently underwent endovascular treatment.	Clinician letter
Left cerebellar cavernoma with previous perinidal haemorrhage	No immediate action. Will have follow-up imaging at phase 2 visit.	Study documents
ACOMA aneurysm	Seen by neurosurgery. Had angiogram. To be repeated in 6 months to assess whether any interval change.	Participant
Possible cavernoma left mid cerebellar peduncle	Had contrast CT. Felt to be solitary cavernoma and deemed low risk.	Study documents
Multiple T2 hypointense lesions and possible pontine cavernoma	Given advice regarding BP control and avoidance of blood-thinners.	Study documents
Basilar tip artery aneurysm, partially thrombosed	Seen by neurosurgery. Underwent exploratory endovascular surgery, but no intervention performed. Subsequently died of stroke (aetiology unknown) around 17 months post-visit.	Participant/family
Prominent right thalamostriate vein with smaller adjacent connecting vessels	Seen by neurology. Further imaging confirmed developmental venous anomaly right frontal area and cavernomata. Given advice regarding BP control and avoidance of blood-thinners.	Clinician letter
Asymmetric configuration of pituitary fossa with T2-hyperintense signal on the left, and mild deviation of pituitary stalk to the right	Seen by neurosurgery. Further imaging confirmed small pituitary adenoma, which is being followed up with interval imaging.	Clinician letter
Signal change and mild swelling within medial aspect of right post-central gyrus	Previous breast cancer. Lung lesion on recent CXR. GP informed oncology re. brain scan abnormality, which in clinical context was felt likely to be a metastasis. Subsequently died.	GP/family
Small median mass at outlet of 4th ventricle without mass effect or hydrocephalus	Seen by neurology. Underwent contrast MRI brain and c-spine. Lesion thought to be subependyoma. Being followed up locally.	Clinician letter
Small well circumscribed extra-axial lesion centred on the pontine cistern on the left	Known meningioma. Already under follow-up. No action taken.	Participant

Right frontal parasagittal meningeal sessile mass	Seen by neurology. Contrast MRI confirmed meningioma and follow-up MRI at 1 year showed no interval change.	Clinician letter
Bulky pituitary gland with convex superior border	Seen by neurosurgery. Asymptomatic. Normal pituitary function tests. Baseline and repeat imaging at 1 year showed no interval change. No longer under follow-up.	Participant
Meningioma overlying left cerebellar hemisphere	Seen by neurology. Contrast MRI confirmed meningioma and follow-up MRI at 1 year showed no interval change.	Clinician letter
Right maxillary antrum almost completely filled by retained secretions.	Seen by ENT. As asymptomatic, not felt to require further tests.	Participant
Complete filling left maxillary sinus with expansion of the osteomeatal complex, raising possibility of an underlying obstructing lesion	Seen by ENT. Underwent nasoendoscopy – no underlying structural lesion. Advised nasal irrigation to decrease congestion. Discharged.	Participant
Complete opacification of the right maxillary sinus and adjacent nasal cavity	Seen by ENT. Had an MRI – no evidence of obstructing lesion. Prescribed antibiotics for sinusitis. Discharged.	Participant
Well circumscribed T1-hyperintense lesion within mandible on right, suggestive of a keratocystic odontogenic tumour	Seen by maxillofacial surgery. Tumour excised.	Participant
T1-hyperintense lesion within suprasellar cistern, differential of which includes dermoid, craniopharyngioma or ACOM aneurysm	Seen by neurology. Further imaging suggested lipoma or small dermoid cyst. No further action recommended.	Clinician letter
