# Supplementary material for: Incidental findings on brain imaging and blood tests: results from the first phase of Insight 46, a prospective observational substudy of the 1946 British birth cohort
Source: BMJ Open. 2019 Jul 31;9(7):e029502. doi: 10.1136/bmjopen-2019-029502 (PMC6678011; doi:10.1136/bmjopen-2019-029502)
Supplement: Author's manuscript [file bmjopen-2019-029502.draft_revisions.pdf]

BMJ Open

BMJ Open is committed to open peer review. As part of this commitment we make the peer review history of every article we publish publicly available.

When an article is published we post the peer reviewers' comments and the authors' responses online. We also post the versions of the paper that were used during peer review. These are the versions that the peer review comments apply to.

The versions of the paper that follow are the versions that were submitted during the peer review process. They are not the versions of record or the final published versions. They should not be cited or distributed as the published version of this manuscript.

BMJ Open is an open access journal and the full, final, typeset and author-corrected version of record of the manuscript is available on our site with no access controls, subscription charges or pay-per-view fees (http://bmjopen.bmj.com).

If you have any questions on BMJ Open's open peer review process please email info.bmjopen@bmj.com

BMJ Open

Incidental findings on brain imaging and blood tests: results from the first phase of Insight 46, a longitudinal prospective sub-study of the 1946 British birth cohort

Journal:	*BMJ Open*
Manuscript ID	bmjopen-2019-029502
Article Type:	Research
Date Submitted by the Author:	29-Jan-2019
Complete List of Authors:	Keuss, Sarah; UCL Queen Square Institute of Neurology, Dementia Research Centre<br>Parker, Thomas; UCL Queen Square Institute of Neurology, Dementia Research Centre<br>Lane, Christopher; UCL Queen Square Institute of Neurology, Dementia Research Centre<br>Hoskote, Chandrashekar; The National Hospital for Neurology and Neurosurgery, Lysholm Department of Neuroradiology<br>Shah, Sachit; The National Hospital for Neurology and Neurosurgery, Lysholm Department of Neuroradiology<br>Cash, David; UCL Queen Square Institute of Neurology, Dementia Research Centre<br>Keshavan, Ashvini; UCL Queen Square Institute of Neurology, Dementia Research Centre<br>Buchanan, Sarah; UCL Queen Square Institute of Neurology, Dementia Research Centre<br>Murray-Smith, Heidi; UCL Queen Square Institute of Neurology, Dementia Research Centre<br>Wong, Andrew; University College London, MRC Unit for Lifelong Health and Ageing<br>James, Sarah-Naomi; University College London, MRC Unit for Lifelong Health and Ageing<br>Lu, Kirsty; UCL Queen Square Institute of Neurology, Dementia Research Centre<br>Collins, Jessica; UCL Queen Square Institute of Neurology, Dementia Research Centre<br>Beasley, Daniel; Kings College London, School of Biomedical Engineering and Imaging Sciences<br>Malone, Ian; UCL Queen Square Institute of Neurology, Dementia Research Centre<br>Thomas, David; UCL Queen Square Institute of Neurology, Leonard Wolfson Experimental Neurology Centre; UCL Queen Square Institute of Neurology, Department of Brain Repair and Neurorehabilitation<br>Barnes, Anna; University College London Hospitals, Institute of Nuclear Medicine<br>Richards, M; University College London, MRC Unit for Lifelong Health and Ageing<br>Fox, Nick; UCL Queen Square Institute of Neurology, Dementia Research Centre

	Schott, Jonathan M.; UCL Queen Square Institute of Neurology, Dementia Research Centre
Keywords:	EPIDEMIOLOGY, INTERNAL MEDICINE, MEDICAL ETHICS, NEUROLOGY, RADIOLOGY & IMAGING

SCHOLARONE™ Manuscripts

Incidental findings on brain imaging and blood tests: results from the first phase of Insight 46, a longitudinal prospective sub-study of the 1946 British birth cohort

Sarah E. Keuss[1]*, Thomas D. Parker[1], Christopher A. Lane[1], Chandrashekar Hoskote[2], Sachit Shah[2], David M. Cash[1], Ashvini Keshavan[1], Sarah M. Buchanan[1], Heidi Murray-Smith[1], Andrew Wong[3], Sarah-Naomi James[3], Kirsty Lu[1], Jessica Collins[1], Daniel G. Beasley[4], Ian B. Malone[1], David L. Thomas[5,6], Anna Barnes[7], Marcus Richards[3]†, Nick C. Fox[1]†, Jonathan M. Schott[1]†

† Joint senior author

*Corresponding author

Dr Sarah E Keuss

Dementia Research Centre

UCL Queen Square Institute of Neurology

Box 16, Queen Square, London WC1N 3BG

s.keuss@ucl.ac.uk | + 44 (0) 20 3448 3193

1. Dementia Research Centre, Queen Square Institute of Neurology, University College London, London, UK
2. Lysholm Department of Neuroradiology, The National Hospital for Neurology and Neurosurgery, Queen Square, London
3. MRC Unit for Lifelong Health and Ageing at UCL, London, UK
4. School of Biomedical Engineering and Imaging Sciences, Kings College, London

For peer review only

5. Leonard Wolfson Experimental Neurology Centre, Queen Square Institute of Neurology, University College London, London, UK
6. Neuroradiological Academic Unit, Department of Brain Repair and Rehabilitation, Queen Square Institute of Neurology, University College London, London, UK
7. Institute of Nuclear Medicine, University College London Hospitals, London, UK

Word count: 3296

For peer review only

ABSTRACT

Objective: To summarise the incidental findings detected on brain imaging and blood tests during the first wave of data collection for the Insight 46 study.

Participants: 502 individuals were recruited from the MRC National Survey of Health and Development (NSHD), the 1946 British birth cohort, based on pre-specified eligibility criteria; mean age was 70.7 (+/-0.7) and 49% were female.

Outcome measures: Data regarding the number and types of incidental findings were summarised as counts and percentages, and 95% confidence intervals were calculated.

Results: 93.8% of participants completed a scan (n=471); 4.5% of scanned participants had a pre-defined reportable abnormality on brain MRI (n=21); suspected vascular malformations and intracranial mass lesions were present in 1.9% (n=9) and 1.5% (n=7) respectively; cerebral aneurysms were the single most common vascular abnormality, affecting 1.1% of participants (n=5), and meningiomas were the most common intracranial lesion, affecting 0.6% of participants (n=3); 34.8% of participants had at least one abnormality on clinical blood tests (n=171), but few reached the pre-specified threshold for urgent action (n=11).

Conclusions: In older adults, aged 69-71 years, potentially serious MRI brain findings were detected in around 5% of participants, and clinical blood test abnormalities were present in

For peer review only

around one third of participants. Knowledge of the expected prevalence of incidental findings in the general population at this age is useful in both research and clinical settings.

STRENGTHS AND LIMITATIONS

- A large number of participants underwent blood testing and brain imaging, at an almost identical age, according to a pre-specified standardised protocol.
- Participants were recruited from the 1946 British birth cohort, a broadly representative sample of the population born in mainland Britain in one week in March 1946.
- Participant perception regarding the disclosure of incidental findings was not formally assessed, nor was the impact on their longer-term health and psychological wellbeing.

For peer review only

INTRODUCTION

Incidental clinical findings are often discovered during the course of conducting research. An incidental finding can be defined as “a finding concerning an individual research participant that has potential health or reproductive importance…but is beyond the aims of the study.”[1] The primary aim of most research is to generate data and advance knowledge, rather than to diagnose health problems in participants, and there is currently no legal requirement for researchers in the UK to report incidental findings to participants.[2] There are, however, important ethical reasons for disclosing certain incidental findings to participants in appropriate circumstances, particularly when they relate to serious and potentially treatable conditions. It is therefore important that studies have protocols in place for managing them. While there is no consensus on how this should be done, it is recommended that researchers weigh up the potential benefits and harm to participants of being informed, as well as considering the associated time and cost, both to the study and to publicly-funded health services.[2]

Incidental findings often lead to anxiety and have the potential to lead to unnecessary and invasive procedures for study participants. Knowledge of the expected prevalence of incidental findings, based on clearly defined protocols for their determination, is important, allowing researchers to be better prepared for managing them, and enabling study participants to be appropriately informed as part of the consent process. Given the increasing use of

For peer review only

neuroimaging in primary, secondary and tertiary care, such information is also useful in the clinical setting, where it can facilitate management decisions. For example, knowing the probability of detecting an abnormality unrelated to a patient's symptoms might influence a clinician's decision to recommend a brain scan in a patient presenting with a benign-sounding headache, or prompt discussion with the patient regarding the pros and cons of scanning.

The Medical Research Council (MRC) National Survey for Health and Development (NSHD) recruited 5362 individuals born in England, Scotland and Wales during the same week in 1946, and has followed them since birth, with over 2500 participants remaining in active follow up.[3] Insight 46 is a longitudinal neuroimaging sub-study of 502 MRC NSHD participants, which aims to investigate genetic and life course factors that contribute to healthy and pathological brain ageing, in particular cerebrovascular and Alzheimer's disease. It involves detailed clinical phenotyping, brain magnetic resonance imaging (MRI), cerebral ß-amyloid positron emission tomography (PET), and blood and urine collection, at two time points approximately two years apart. The full study protocol, which includes clear criteria for reporting incidental findings, has been described elsewhere.[4]

The aim of this study is to summarise the incidental findings detected on brain imaging and blood tests during the first wave of data collection for Insight 46. Several studies have reported rates of incidental findings in different samples previously,[5-10] but to our knowledge, none have reported on findings from a representative country-wide birth cohort with pre-specified standardised protocols in place.

METHODS

For peer review only

Recruitment

Individuals were recruited from NSHD participants who attended a study visit at age 60-64, who had previously indicated that they would be willing to consider participating in a study visit in London, and for whom relevant life course data was available. NSHD participants who met these criteria were sent an information booklet about the study and then recruited by a study doctor via telephone. Those with known contraindications to PET or MRI scanning were not recruited. Eligibility criteria were relaxed towards the end of the study, allowing inclusion of some individuals with a few missing life course data-points, in order to achieve the study's recruitment target.

Consent

The booklet sent to participants prior to their visit contained a detailed description of the study tests, including information about the study protocol with regard to incidental findings. All participants provided written consent to participate. They could choose to opt out of receiving correspondence about blood results but had to consent to their general practitioner (GP) being informed about them.

Neuroimaging

Participants underwent brain imaging on a single Biograph mMR 3 Tesla PET/MRI scanner (Siemens Healthcare). Participants were injected via an intravenous cannula with the ^{18}F amyloid PET ligand Florbetapir at the start of the imaging session, and dynamic amyloid data was obtained over 60 minutes. MRI data was acquired simultaneously, including: volumetric

For peer review only

T1-weighted, T2-weighted and FLAIR sequences; resting state functional MRI; multi-shell diffusion-weighted imaging; 3D gradient echo sequence for T2*-weighted/ susceptibility-weighted imaging; and arterial spin labelling (non-invasive perfusion imaging).

Blood tests

Participants provided blood samples for standard clinical tests including haemoglobin, platelet count, vitamin B12, urea, creatinine, random glucose and thyroid stimulating hormone (TSH). Samples were also taken for biomarker and genetic testing. Results of the clinical blood tests were reported back to the study team via email within 24 hours. Samples for biomarker and genetic testing were stored for future analysis.

Duty of care protocol for neuroimaging

All T1-weighted, T2-weighted and FLAIR MRI sequences were reviewed by one of two consultant neuroradiologists within two weeks of the scan. Other sequences were not routinely reviewed, on the basis that they do not form part of a standard diagnostic MRI examination in clinical practice. Neuroradiologists used a list of pre-specified reportable and non-reportable abnormalities to flag scans as being potentially reportable (Table 1). This list was adapted from the UK Biobank study, which classified findings as reportable if they were potentially serious (i.e. life-threatening or likely to have a major impact on quality of life or function).[11] Neuroradiologists were also encouraged to flag scans with other unexpected findings if there was any possibility that further assessment might be required.

Reportable findings	Non-reportable findings

For peer review only

▪ Acute brain infarction<br>▪ Acute brain haemorrhage (note: not old bleeds)<br>▪ Intracranial mass lesions (note: not meningiomas in locations considered unlikely to cause problems)<br>▪ Suspected intracranial aneurysm or vascular malformation (including cavernomata) (note: not aneurysms <7mm in diameter)<br>▪ Colloid cyst of the 3rd ventricle<br>▪ Acute hydrocephalus<br>▪ Significant sinus disease with suspicion of underlying pathology (e.g. unilateral sinus opacification)<br>▪ Other unexpected, serious, or life-threatening findings	▪ White matter hyperintensities<br>▪ Suspected demyelination<br>▪ Non-acute brain infarction<br>▪ Chronic hydrocephalus<br>▪ Asymmetric ventricles<br>▪ Lipoma of the corpus callosum<br>▪ Developmental abnormalities<br>▪ Enlarged perivascular spaces<br>▪ Chiari malformation<br>▪ Hippocampal or other focal atrophy

Table 1. List of reportable and non-reportable MRI abnormalities.

The reporting process was performed electronically using the web-based data management tool XNAT (www.xnat.org), thereby providing an audit trail (Figure 1). Reporting radiologists downloaded images from the XNAT server, reviewed them, and then completed a radiological read report within XNAT. This took around ten minutes per scan. If a scan was flagged as potentially reportable, the study coordinator was automatically notified, and a multidisciplinary meeting was organised within four weeks of the study visit. The reporting neuroradiologist, study chief investigator, and other relevant members from the study team were present at this meeting. If the abnormality was agreed to meet criteria for being reportable, the team decided on a clinical action plan (e.g. further imaging and/or specialist referral). A study doctor then contacted the participant and their GP, by telephone and in writing, providing them with information about the MRI abnormality and the recommended clinical action. Since data was collected in an anonymised form, it was not possible to share the images for clinical use.

For peer review only

Results of the amyloid PET scan were not fed back to participants because of the diagnostic and prognostic uncertainties of using this test in cognitively normal individuals, and lack of disease-modifying treatments for people with amyloid pathology. These ethical considerations have been discussed elsewhere.[12]

Duty of care protocol for blood tests

Results of the clinical blood tests were reviewed by the study doctor and reported back to the participant and their GP in writing within two weeks of the study visit. If results fell outside the normal reference range (Table 2), participants were advised in writing to discuss this with their GP. If results were deemed to be significantly abnormal, falling beyond pre-specified urgent action levels (Table 2), the study doctor contacted the participant and their GP by telephone within 48 hours of the study visit. These pre-specified levels were adapted from those used at the NSHD whole cohort sweep at age 60-64.[3] They reflect values at which urgent action would be warranted in clinical practice and were developed in consultation with clinical scientists and physicians in the relevant field. Biomarker and genetic test results were not reported back to participants.

For peer review only

Blood test	Normal reference range	Urgent action level
Haemoglobin (male)	13.0-17.0 g/dl	<10 or >20 g/dl
Haemoglobin (female)	11.5-15.5 g/dl	<10 or >20 g/dl
Platelets	150-400 10^9/l	<100 or >1000 10^9/l
Vitamin B12	191-900 pg/ml	<100 pg/ml
Urea	1.7-8.3 mmol/l	>20 mmol/l
Creatinine (male)	66-112 µmol/l	>200 µmol/l
Creatinine (female)	49-92 µmol/l	>200 µmol/l

For peer review only - http://bmjopen.bmj.com/site/about/guidelines.xhtml

Glucose	3.5-10 mmol/l	>20 mmol/l
Thyroid stimulating hormone	0.27-5.5 mIU/l	<0.1 or >10 mIU/l

Table 2. Clinical blood tests, their normal reference ranges, and urgent action levels

Analysis

Data regarding the number and types of incidental findings, and the actions taken by the study team in response to them, were summarised as counts and percentages, and 95% confidence intervals (CIs) for proportions were calculated. Sex differences were assessed using a two-sample test of proportions in STATA version 14.2. Estimated glomerular filtration rate (eGFR) was derived using the Modification of Diet in Renal Disease (MDRD) study equation: GFR (ml/min/1.73m^2) = 175 x (Scr/88.4)$^{-1.154}$ x (Age)$^{-0.203}$ (x 0.742 if female) where Scr equals serum creatinine in µmol/L.

Participant involvement

Study members helped in the design of the Insight 46 study through participation in focus groups. Participants were invited to complete evaluation forms following their study visit, outlining any positive or negative aspects of their experience. Results from the Insight 46 study will be disseminated to participants through newsletters and public engagement events.

RESULTS

502 participants attended a study visit in London from throughout mainland Britain between May 2015 and January 2018. Mean age was 70.7 (+/-0.7) years and 49% were female.

For peer review only

Brain MRI

93.8% of participants completed a brain scan (n=471). The most common reason for non-completion was claustrophobia (n=25). Other reasons included: being unable to lie comfortably in the scanner (n=3); concerns about radiation (n=1); possible metallic implants (n=1); and withdrawal from the study (n=1). 7.6% of scans (n=36) were flagged by neuroradiologists as having potentially reportable abnormalities for review. Following discussion between the reporting neuroradiologist and study chief investigator, 58.3% of these scans (n=21) were deemed to have an abnormality that fulfilled criteria for being reportable. Therefore, in total, 4.5% of all scans had an incidental finding that was reported to the participant and their GP.

Table 3 summarises the number and percentage of reportable MRI abnormalities by type and sex. Females were more likely to have a reportable MRI abnormality than males (6.5% vs 2.5%; p=0.034). The most common abnormalities were suspected vascular malformations and intracranial mass lesions, which were detected in 1.9% (n=9) and 1.5% (n=7) of participants respectively. Cerebral aneurysms were the most common vascular abnormality, affecting 1.1% of participants (n=5; Figure 2A). Meningiomas were the most common intracranial lesion, affecting 0.6% of participants (n=3; Figure 2B).

With regards to management of incidental findings, further imaging was recommended in 66.6% of cases (n=14); specialist referral was advised in 57.1% of cases (n=12); advice regarding medication and management was given in 19% of cases (n=4); and no action was recommended in 9.5% of cases where the abnormalities were found to be pre-existing and already being managed by the participant's local health services (n=2). All aneurysms not

For peer review only

previously known about were referred for an expedited neurosurgical opinion (n=4). In one participant, the neurosurgeon felt that further investigation was not warranted, because the aneurysm was small and unlikely to require any intervention. The other three participants underwent dedicated vascular imaging with contrast. Two of these participants are being followed up with interval imaging, and one participant had their warfarin stopped and was referred for surgery. Of the meningioma cases, one was already known about and being followed up locally. The other two participants were referred to a neurologist and underwent further imaging with gadolinium contrast to confirm the diagnosis, before being followed up with interval imaging.

For peer review only

	All (Total = 471)		Male (Total = 241)		Female (Total = 230)	
	Number	% (95% CI)	Number	% (95% CI)	Number	% (95% CI)
Any abnormality	21	4.5 (2.9, 6.8)	6	2.5 (1.1, 5.5)	15	6.5 (4.0, 10.6)
Acute brain infarction	-	-	-	-	-	-
Acute brain haemorrhage	-	-	-	-	-	-
Suspected intracranial mass lesion	7	1.5 (0.7, 3.1)	2	0.8 (0.2, 3.3)	5	2.2 (0.9, 5.1)
Suspected intracranial aneurysm or vascular malformation	9	1.9 (1.0, 3.6)	2	0.8 (0.2, 3.3)	7	3.0 (1.4, 6.3)
Colloid cyst of the 3rd ventricle	-	-	-	-	-	-
Acute hydrocephalus	-	-	-	-	-	-
Significant sinus pathology	3	0.6 (0.2, 2.0)	1	0.4 (0.1, 2.9)	2	0.9 (0.2, 3.4)
Other*	2	0.4 (0.1, 1.7)	1	0.4 (0.1, 2.9)	1	0.4 (0.6, 3.1)

* possible keratocystic odontogenic tumour of right mandible (n=1); hyperintense area in the suprasellar cistern with differential diagnosis of small dermoid cyst, craniopharyngioma, or thrombosed anterior communicating artery aneurysm (n=1)

Table 3. Number and percentage of reportable MRI abnormalities by type and sex

For peer review only

Standard clinical blood tests

Venepuncture was successful in over 99% of participants (n=498). There were missing blood result values in some participants (n=9) due to insufficient samples, lab errors, clumped platelets or a clotted sample. 34.8% of participants had at least one abnormality on standard clinical blood tests (n=171). Of those participants with abnormalities, urgent action was required for 6.5% (n=11). In many of these cases (n=6), the participant's GP confirmed that the abnormality was pre-existing and already being managed. Table 4 summarises the number and percentage of blood test abnormalities by type and sex. Males were significantly more likely to have at least one blood test abnormality than females (41.3% vs 28.0%; p=0.002). Almost all participants chose to receive a copy of their clinical blood test results (n=496).

For peer review only

	All (Total = 498)		Male (Total = 255)		Female (Total = 243)	
	Number	% (95% CI)	Number	% (95% CI)	Number	% (95% CI)
Any abnormality	171/491	34.8 (30.7, 39.2)	104/252	41.3 (35.3, 47.5)	67/239	28.0 (22.7, 34.1)
Polycythaemia	15/494	3.0 (1.8, 5.0)	11/254	4.3 (2.4, 7.7)	4/240	1.7 (0.6, 4.4)
Anaemia	19/494	3.8 (2.5, 6.0)	14/254	5.5 (3.3, 9.1)	5/240	2.1 (0.9, 5.0)
Thrombocytosis	10/492	2.0 (1.1, 3.7)	2/252	0.8 (0.2, 3.1)	8/240	3.3 (1.7, 6.6)
Thrombocytopenia	11/492	2.2 (1.2, 4.0)	9/252	3.6 (1.9, 6.7)	2/240	0.8 (0.2, 3.3)
Elevated vitamin B12	10/495	2.0 (1.1, 3.7)	5/253	2.0 (0.8, 4.7)	5/242	2.1 (0.9, 4.9)
Low vitamin B12	16/495	3.2 (2.0, 5.2)	6/253	2.4 (1.1, 5.2)	10/242	4.1 (2.2, 7.5)
Elevated urea	40/497	8.0 (6.0, 10.8)	23/254	9.1 (6.1, 13.3)	17/243	7.0 (4.4, 11.0)
Elevated creatinine	17/497	3.4 (2.1, 5.4)	10/254	3.9 (2.1, 7.2)	7/243	2.9 (1.4, 5.9)
Low creatinine	41/497	8.2 (6.1, 11.0)	33/254	13.0 (9.4, 17.7)	8/243	3.3 (1.6, 6.5)
eGFR<60 *	43/497	8.7 (6.5, 11.5)	15/254	5.9 (3.6, 9.6)	28/243	11.5 (8.1, 16.2)
Hyperglycaemia	21/497	4.2 (2.8, 6.4)	16/254	6.3 (3.9, 10.0)	5/243	2.1 (0.9, 4.9)
Hypoglycaemia	5/497	1.0 (0.4, 2.4)	1/254	0.4 (0.1, 2.8)	4/243	1.6 (0.6, 4.3)
Elevated TSH	13/496	2.6 (1.5, 4.5)	4/253	1.6 (0.6, 4.2)	9/243	3.7 (1.9, 7.0)
Low TSH	9/496	1.8 (0.9, 3.6)	-	-	9/243	3.7 (1.9, 7.0)
Urgent action	11/489	2.2 (1.2, 4.0)	3/250	1.2 (0.4, 3.7)	8/239	3.3 (1.7, 6.6)

Table 4. Number and percentage of clinical blood test abnormalities by type and sex

*eGFR (ml/min/1.73m^2) was calculated for this analysis to facilitate comparison with other studies; it was not reported back to participants

For peer review only

DISCUSSION

In this study of older adults, aged 69-71, reportable incidental findings on brain MRI were present in 4.5% of participants, with suspected vascular malformations and intracranial mass lesions present in 1.9% and 1.5% of participants respectively. Clinical blood test abnormalities were common, affecting around one third of participants. However, very few blood test abnormalities required urgent action, and many of those that did were previously known to the participants' GPs and had already been acted upon.

Comparison with other studies

Due to the recent proliferation of neuroimaging research, incidental findings on brain MRI are often reported in the literature.[5-10] The reported prevalence varies between studies, likely reflecting differences in the definition of what constitutes an incidental finding, as well as variability in participant demographics and imaging protocols. Most imaging studies, for example, do not require routine review of all scans by a radiologist.[13] Often, researchers will only ask for a radiologist opinion if an abnormality is identified incidentally by a radiographer during scanning or by researchers during data analysis.

A 2018 systematic review reported an overall prevalence of 1.4% (95% CI 1.0% to 2.1%) for potentially serious brain incidental findings.[14] This is somewhat lower than the 4.5% (95% CI 2.9% to 6.8%) detected in Insight 46 participants, although this review consisted mainly of studies with younger participants using scanners of 1.5 Tesla or less. The 1936 Lothian Birth Cohort (LBC) reported an overall prevalence of 32% (95% CI 28% to 35%) in their participants at age 73.[5] This higher rate is likely due to their inclusion of old stroke lesions, developmental

For peer review only

anomalies and benign pathologies, which were not deemed to be reportable in the Insight 46 study. Indeed, when comparing specific abnormalities, namely intracranial mass lesions and vascular malformations, rates were very similar between the LBC and Insight 46 study, i.e. 1.4% (95% CI 0.7% to 2.6%) and 2% (95% CI 1.1% to 3.3%) respectively in LBC subjects, compared with 1.5% (95% CI 0.7% to 3.1%) and 1.9% (95% CI 1.0% to 3.6%) in Insight 46 participants. Another large population-based study, which included over 2000 subjects aged 45 to 97 years old, found similar results, with a prevalence of 1.6% (95% CI 1.1% to 2.2%) for benign intracranial mass lesions, and 1.8% (95% CI 1.2% to 2.4%) for cerebral aneurysms.[6]

With regards to blood tests, the Insight 46 study tended to have either similar or lower rates of abnormalities than other studies. The prevalence of anaemia in a systematic review of studies involving community-dwelling older adults was 12%, which is somewhat higher than the 3.8% (95% CI 2.5% to 6.0%) detected in Insight 46 participants.[15] The prevalence of chronic kidney disease (CKD) stages 3-5 (eGFR <60ml/min/1.73m^2) is estimated to be around 6.1% in adults under 65 in England, rising to 13.5% for individuals aged 65-74, according to data collected in 2009-10 Health Survey for England and 2011 Census.[16] This is broadly in keeping with the rate of 8.7% (95% CI 6.5% to 11.5%) detected in Insight 46 participants. Vitamin B12 deficiency was detected in around 5% of individuals aged 65-74 years old in a large UK-based study, compared with 3.2% (95% CI 2.0% to 5.2%) in Insight 46 participants.[17] Another large UK-based study found a prevalence of 7.9% (95% CI 6.4% to 9.6%) for elevated TSH and 6.0% (95% CI 4.7% to 7.4%) for low TSH in adults over 60 years old, somewhat higher than the 2.6% (95% CI 1.5% to 4.5%) and 1.8% (95% CI 0.9% to 3.6%) detected in Insight 46 participants.[18]

For peer review only

Discrepancies in the reported prevalence of blood test abnormalities between Insight 46 and other studies may be partly related to differences in laboratory assays, thresholds for defining abnormal values, and participant demographics. However, it also likely that certain blood test abnormalities are under-represented in Insight 46, since participants underwent clinical blood testing at a previous study visit aged 60-64 years old, and any abnormalities detected then were likely addressed at that time.[19] Indeed, comparing participant results at age 60-64 with those in the Insight 46 study revealed that: only 2 out of 9 participants still had anaemia; 8 out 27 still had an elevated TSH; and 5 out of 10 still had a low TSH.

Strength and weaknesses

A major strength of the Insight 46 study is that it involved a large number of participants who underwent brain imaging and blood testing, at an almost identical age, according to a pre-specified standardised protocol. These participants were all recruited from the NSHD, the 1946 British birth cohort, a broadly representative sample of the population born in mainland Britain in 1946. High resolution MRI sequences were obtained using the same PETMR scanner, and images were systematically reviewed by two experienced consultant neuroradiologists. This process was user-friendly and automated where possible, allowing scans to be reported within a short timeframe, thereby reducing the workload of the neuroradiologists.

The duty of care protocol was developed in accordance with the MRC and Wellcome Trust framework on management of health-related research findings.[2] Any potentially serious brain MRI findings or blood test abnormalities were reported back to participants and their GPs, in keeping with the ethical principle of beneficence. Findings were not disclosed if tests lacked clinical utility or were not actionable, in order to minimise participant distress and harm.

For peer review only

Participants were fully informed of the protocol for managing incidental findings as part of the consent process and were given the choice regarding whether they wanted to receive a copy of their blood results, thereby respecting their autonomy to make decisions about their own health.

A limitation of this study is that participant perception regarding the disclosure of incidental findings was not formally assessed, nor was the impact on their longer-term health and psychological wellbeing. Many participants, however, gave informal feedback on post-visit evaluation forms that they appreciated being told about findings pertinent to their health and saw this as a benefit of being involved in the study. Moreover, almost all participants chose to receive a copy of their blood test results. These observations are consistent with results of a study commissioned by the Wellcome Trust and MRC, which found overwhelming public support for the disclosure of incidental findings in research, particularly in relation to serious and treatable conditions.[20]

Implications and future work

The findings of this study will be relevant to future studies involving older adults, including clinical trials of secondary prevention drugs for Alzheimer's disease, which often involve MRI-based outcome measures and blood monitoring. By defining what is actionable and providing the expected prevalence of incidental findings on brain MRI and clinical blood tests in this age group, researchers may be better prepared for managing incidental findings, and participants better informed of their likelihood as part of the consent process.

The findings also have implications for clinical practice. In patients with benign-sounding headaches and normal neurological examination, for example, the likelihood of detecting a

For peer review only

serious intracranial cause on brain imaging is less than 1%.[21,22] Nonetheless, patients presenting with chronic headache frequently undergo brain imaging, usually to provide reassurance, and often at the patient's own request. These patients are rarely consented for the risk of discovering an incidental finding, despite the potential negative consequences. Greater awareness of the expected frequency and nature of incidental findings on brain imaging and blood tests should allow clinicians to counsel patients regarding their probability, and to balance this risk against the potential benefits of undergoing a test when deciding whether it is appropriate.

Further work is needed to assess the implications of disclosing incidental findings, including the psychological effects and longer-term clinical consequences, as well as the impact on research integrity, particularly in longitudinal population studies where disclosure might lead to a biased sample.

For peer review only

FIGURE LEGENDS

Figure 1. Simplified overview of the process for viewing and reporting scans using XNAT

Figure 2. (A) Sagittal T1-weighted image, demonstrating a 10mm aneurysm (arrow) arising from tip of the basilar artery. (B) Coronal FLAIR image, demonstrating a broad-based extra-axial lesion (asterisk) overlying the right superior frontal gyrus, consistent with a meningioma.

For peer review only

STATEMENTS

Funding

Insight 46 is funded by grants from Alzheimer's Research UK (ARUK-PG2014–1946, ARUK-PG2017-1946 PIs Schott, Fox, Richards), the Medical Research Council Dementias Platform UK (CSUB19166 PIs Schott, Fox, Richards), the Wolfson Foundation (PR/ylr/18575 PIs Fox, Schott), the Medical Research Council (MC_UU_12019/1 PI Kuh and MC_UU_12019/3 PI Richards), the Wellcome Trust (Clinical Research Fellowship 200,109/Z/15/Z Parker) and Brain Research Trust (UCC14191, PI Schott). AVID Radiopharmaceuticals (a wholly owned subsidiary of Eli Lilly) provide the PET amyloid tracer (Florbetapir) but had no part in the design of the study.

Competing interests

NCF's research group has received payment for consultancy or for conducting studies from Avid Radiopharmaceuticals, Biogen, Eisai, Elan, Eli Lilly Research Laboratories, GE Healthcare, IXICO, Janssen, Johnson & Johnson, Lundbeck, Pfizer, Roche, Sanofi-Aventis and Wyeth Pharmaceuticals. NCF receives no personal compensation for the activities mentioned above. JMS has received research funding from Avid Radiopharmaceuticals (a wholly owned

For peer review only

subsidiary of Eli Lilly), has consulted for Roche Pharmaceuticals, Biogen, and Eli Lilly, given educational lectures sponsored by GE, Eli Lilly and Biogen, and serves on a Data Safety Monitoring Committee for Axon Neuroscience SE.

Ethics

Ethical approval was granted by the National Research Ethics Service (NRES) Committee London (REC reference 14/LO/1173, PI Schott).

Author Contributions

SEK and JMS conceived the manuscript. TDP, CAL, AK, SEK, SMB, HMS and AW recruited participants to the study. CH and SS reviewed and reported the MRI brain scans. TDP, CAL, AK, SEK, SMB, SNJ, KL and JC contributed to data collection. DMC, IBM, DLT and AB were responsible for setting up the imaging acquisition protocols, image processing and quality control. DGB was involved in data management. SEK analysed the data and drafted the initial manuscript. JMS, NCF and MR are Co-Principal Investigators of the study. All authors critically revised the manuscript and approved the submitted version.

Acknowledgements

We are very grateful to those study members who helped in the design of the study through focus groups, and to the participants both for their contributions to Insight 46 and for their commitment to research over the last seven decades. We are grateful to the radiographers and nuclear medicine physicians (Professor Ashley Groves, Dr Jamshed Bomanji, Dr Irfan Kayani) at the UCL Institute of Nuclear Medicine, and to

For peer review only

the staff at the Leonard Wolfson Experimental Neurology Centre at UCL. We would like to acknowledge Dan Marcus and Rick Herrick for assistance with XNAT, Dr Philip Curran for assistance with data sharing with the MRC Unit for Lifelong Health and Ageing, the DRC trials team for assistance with imaging QC, Mark White for his work on data connectivity, and Suzie Barker for her assistance with research governance.

For peer review only

REFERENCES

1. Wolf FM, Lawrenz FP, Nelson CA, et al. Managing incidental findings in human subjects research: analysis and recommendations. *J Law Med Ethics* 2008;36:219-211.
2. Medical Research Council, The Wellcome Trust. Framework on the feedback of health-related findings in research 2014. URL: https://www.mrc.ac.uk/documents/pdf/mrc-wellcome-trust-framework-on-the-feedback-of-health-related-findings-in-researchpdf/ [date accessed – 25th January 2019].
3. Kuh D, Pierce M, Adams J, et al. Cohort Profile: Updating the cohort profile for the MRC National Survey of Health and Development: a new clinic-based data collection for ageing research. *Int J Epidemiol* 2011;40:e1-e9.

4. Lane CA, Parker TP, Cash DM, et al. Study protocol: Insight 46 – a neuroscience sub-study of the MRC National Survey for Health and Development. *BMC Neurol* 2017;17:75.

5. Sandeman EM, Hernandez Mdel C, Morris Z, et al. Incidental Findings on Brain MR Imaging in Older Community-Dwelling Subjects Are Common but Serious Medical Consequences Are Rare: A Cohort Study. *PLoS ONE* 2013;8:e71467.

6. Vernooij MW, Ikram MA, Tanghe HL, et al. Incidental findings on brain MRI in the general population. *N Eng J Med* 2007;356:1821-1828.

7. Bos D, Poels MMF, Adams HHH, et al. Prevalence, clinical management, and natural course of incidental findings on brain MR images: the population-based Rotterdam Scan Study. *Radiology* 2016;281:507-515.

8. Yue NC, Longstreth WT Jr, Elster AD, et al. Clinically serious abnormalities found incidentally at MR imaging of the brain: data from the Cardiovascular Health Study. *Radiology* 1997;202:41-46.

9. Katzman GL, Dagher AP, Patronas NJ. Incidental findings on brain magnetic resonance imaging from 1000 asymptomatic volunteers. *JAMA* 1999;282:36-39.

10. Boutet C, Vassal F, Celle S, et al. Incidental findings on brain magnetic resonance imaging in the elderly: the PROOF study. *Brain Imaging Behav* 2017;11:293-299.

11. Gibson LM, Littlejohns TJ, Adamska L, et al. Impact of detecting potentially serious incidental findings during multi-modal imaging. *Wellcome Open Research* 2018;2:114.

12. Harkins K, Sankar P, Sperling P, et al. Development of a process to disclose amyloid imaging results to cognitively normal older adult research participants. *Alzheimers Res Ther* 2015;7:26.

For peer review only

13. Booth TC, Waldman AD, Wardlaw JM, et al. Management of incidental findings during imaging research in “healthy” volunteers: current UK practice. *Br J Radiol* 2012;85:11-21.

14. Gibson LM, Paul L, Chappell F, et al. Potentially serious incidental findings on brain and body magnetic resonance imaging of apparently asymptomatic adults: systematic review and meta-analysis. *BMJ* 2018;363:k4577.

15. Gaskell H, Derry S, Moore RA, McQuay HJ. Prevalence of anaemia in older persons: systematic review. *BMC Geriatr* 2008;8:1.

16. Public Health England. Chronic kidney disease prevalence model. 2014. URL: https://assets.publishing.service.gov.uk/government/uploads/system/uploads/attachment_data/file/612303/ChronickidneydiseaseCKDprevalencemodelbriefing.pdf [date accessed - 25th January 2019].

17. Clarke R, Grimley EJ, Schneede J, et al. Vitamin B12 deficiency and folate deficiency in later life. *Age Ageing* 2004;33:34-41.

18. Parle JV, Franklyn JA, Cross KW, et al. Prevalence and follow-up of abnormal thyrotrophin (TSH) concentrations in the elderly in the United Kingdom. *Clin Endocrinol* 1991;34:77-83.

19. Pierce MB, Silverwood RJ, Nitsch D, et al. Clinical disorders in a post war British cohort reaching retirement: evidence from the first national birth cohort. *PLoS ONE* 2012;7:e44857

20. Opinion Leader, Wellcome Trust, Medical Research Council. Assessing public attitudes to health-related findings in research 2012. URL: https://wellcome.ac.uk/sites/default/files/wtvm055196_0.pdf [date accessed - 25th January 2019].

For peer review only

21. Sempere AP, Porta-Etessam J, et al. Neuroimaging in the evaluation of patients with non-acute headache. *Cephalgia* 2005;5:30-35.

22. Detsky ME, Mcdonald DR, et al. Does the patient with headache have a migraine or need neuroimaging? *JAMA* 2006;296:1274-83.

For peer review only

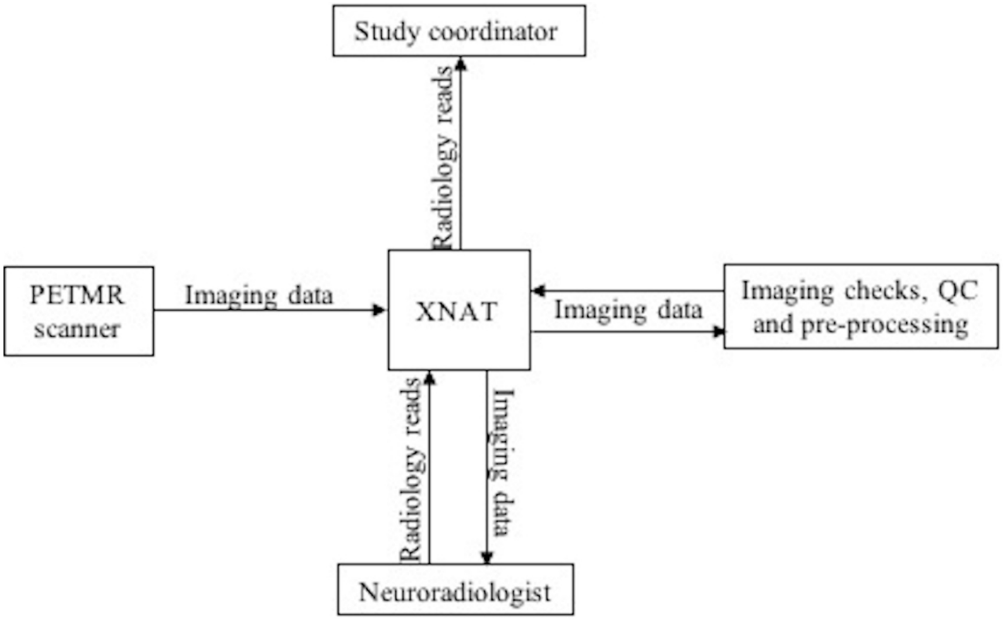

Figure 1. Simplified overview of the process for viewing and reporting scans using XNAT

147x91mm (300 x 300 DPI)

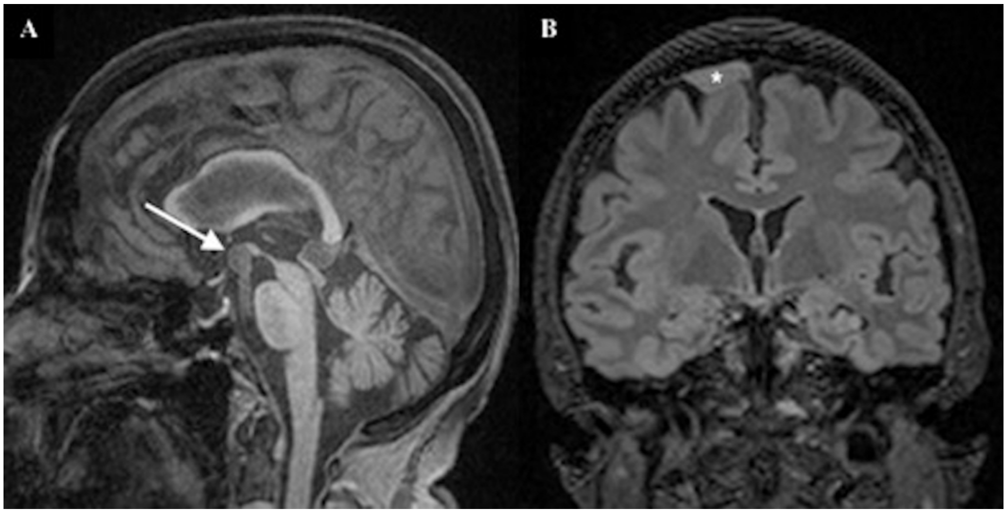

Figure 2. (A) Sagittal T1-weighted image, demonstrating a 10mm aneurysm (arrow) arising from tip of the basilar artery. (B) Coronal FLAIR image, demonstrating a broad-based extra-axial lesion (asterisk) overlying the right superior frontal gyrus, consistent with a meningioma.

160x81mm (300 x 300 DPI)

STROBE Statement—Checklist of items that should be included in reports of ***cross-sectional studies***

	Item No	Recommendation	Page No
**Title and abstract**	1	(*a*) Indicate the study's design with a commonly used term in the title or the abstract	1
		(*b*) Provide in the abstract an informative and balanced summary of what was done and what was found	3
**Introduction**			
Background/rationale	2	Explain the scientific background and rationale for the investigation being reported	5
Objectives	3	State specific objectives, including any prespecified hypotheses	6
**Methods**			
Study design	4	Present key elements of study design early in the paper	6
Setting	5	Describe the setting, locations, and relevant dates, including periods of recruitment, exposure, follow-up, and data collection	11
Participants	6	(*a*) Give the eligibility criteria, and the sources and methods of selection of participants	6,7
Variables	7	Clearly define all outcomes, exposures, predictors, potential confounders, and effect modifiers. Give diagnostic criteria, if applicable	8
Data sources/ measurement	8*	For each variable of interest, give sources of data and details of methods of assessment (measurement). Describe comparability of assessment methods if there is more than one group	7-10
Bias	9	Describe any efforts to address potential sources of bias	N/A
Study size	10	Explain how the study size was arrived at	6,7
Quantitative variables	11	Explain how quantitative variables were handled in the analyses. If applicable, describe which groupings were chosen and why	
Statistical methods	12	(*a*) Describe all statistical methods, including those used to control for confounding	10
		(*b*) Describe any methods used to examine subgroups and interactions	10
		(*c*) Explain how missing data were addressed	N/A
		(*d*) If applicable, describe analytical methods taking account of sampling strategy	N/A
		(*e*) Describe any sensitivity analyses	N/A
**Results**			
Participants	13*	(a) Report numbers of individuals at each stage of study—eg numbers potentially eligible, examined for eligibility, confirmed eligible, included in the study, completing follow-up, and analysed	11,13
		(b) Give reasons for non-participation at each stage	11,13
		(c) Consider use of a flow diagram	-
Descriptive data	14*	(a) Give characteristics of study participants (eg demographic, clinical, social) and information on exposures and potential confounders	11
		(b) Indicate number of participants with missing data for each variable of interest	11,13
Outcome data	15*	Report numbers of outcome events or summary measures	11-15
Main results	16	(*a*) Give unadjusted estimates and, if applicable, confounder-adjusted estimates and their precision (eg, 95% confidence interval). Make clear which confounders were adjusted for and why they were included	11-15

For peer review only - http://bmjopen.bmj.com/site/about/guidelines.xhtml

		(*b*) Report category boundaries when continuous variables were categorized	N/A
		(*c*) If relevant, consider translating estimates of relative risk into absolute risk for a meaningful time period	N/A
Other analyses	17	Report other analyses done—eg analyses of subgroups and interactions, and sensitivity analyses	N/A
**Discussion**			
Key results	18	Summarise key results with reference to study objectives	16
Limitations	19	Discuss limitations of the study, taking into account sources of potential bias or imprecision. Discuss both direction and magnitude of any potential bias	19
Interpretation	20	Give a cautious overall interpretation of results considering objectives, limitations, multiplicity of analyses, results from similar studies, and other relevant evidence	16,17
Generalisability	21	Discuss the generalisability (external validity) of the study results	17,18
**Other information**			
Funding	22	Give the source of funding and the role of the funders for the present study and, if applicable, for the original study on which the present article is based	22

*Give information separately for exposed and unexposed groups.

Note: An Explanation and Elaboration article discusses each checklist item and gives methodological background and published examples of transparent reporting. The STROBE checklist is best used in conjunction with this article (freely available on the Web sites of PLoS Medicine at http://www.plosmedicine.org/, Annals of Internal Medicine at http://www.annals.org/, and Epidemiology at http://www.epidem.com/). Information on the STROBE Initiative is available at www.strobe-statement.org.

For peer review only

For peer review only - http://bmjopen.bmj.com/site/about/guidelines.xhtml

BMJ Open

Incidental findings on brain imaging and blood tests: results from the first phase of Insight 46, a prospective observational sub-study of the 1946 British birth cohort

Journal:	*BMJ Open*
Manuscript ID	bmjopen-2019-029502.R1
Article Type:	Research
Date Submitted by the Author:	15-Jun-2019

Complete List of Authors:	Keuss, Sarah; UCL Queen Square Institute of Neurology, Dementia Research Centre<br>Parker, Thomas; UCL Queen Square Institute of Neurology, Dementia Research Centre<br>Lane, Christopher; UCL Queen Square Institute of Neurology, Dementia Research Centre<br>Hoskote, Chandrashekar; The National Hospital for Neurology and Neurosurgery, Lysholm Department of Neuroradiology<br>Shah, Sachit; The National Hospital for Neurology and Neurosurgery, Lysholm Department of Neuroradiology<br>Cash, David; UCL Queen Square Institute of Neurology, Dementia Research Centre<br>Keshavan, Ashvini; UCL Queen Square Institute of Neurology, Dementia Research Centre<br>Buchanan, Sarah; UCL Queen Square Institute of Neurology, Dementia Research Centre<br>Murray-Smith, Heidi; UCL Queen Square Institute of Neurology, Dementia Research Centre<br>Wong, Andrew; University College London, MRC Unit for Lifelong Health and Ageing<br>James, Sarah-Naomi; University College London, MRC Unit for Lifelong Health and Ageing<br>Lu, Kirsty; UCL Queen Square Institute of Neurology, Dementia Research Centre<br>Collins, Jessica; UCL Queen Square Institute of Neurology, Dementia Research Centre<br>Beasley, Daniel; Kings College London, School of Biomedical Engineering and Imaging Sciences<br>Malone, Ian; UCL Queen Square Institute of Neurology, Dementia Research Centre<br>Thomas, David; UCL Queen Square Institute of Neurology, Leonard Wolfson Experimental Neurology Centre; UCL Queen Square Institute of Neurology, Department of Brain Repair and Neurorehabilitation<br>Barnes, Anna; University College London Hospitals, Institute of Nuclear Medicine<br>Richards, M; University College London, MRC Unit for Lifelong Health and Ageing<br>Fox, Nick; UCL Queen Square Institute of Neurology, Dementia Research Centre

	Schott, Jonathan M.; UCL Queen Square Institute of Neurology, Dementia Research Centre
**Primary Subject Heading**:	Epidemiology
Secondary Subject Heading:	Research methods, Radiology and imaging, Ethics, Neurology
Keywords:	EPIDEMIOLOGY, INTERNAL MEDICINE, MEDICAL ETHICS, NEUROLOGY, RADIOLOGY & IMAGING

SCHOLARONE™
Manuscripts

Incidental findings on brain imaging and blood tests: results from the first phase of Insight 46, a prospective observational sub-study of the 1946 British birth cohort

Sarah E. Keuss[1], Thomas D. Parker[1], Christopher A. Lane[1], Chandrashekar Hoskote[2], Sachit Shah[2], David M. Cash[1], Ashvini Keshavan[1], Sarah M. Buchanan[1], Heidi Murray-Smith[1], Andrew Wong[3], Sarah-Naomi James[3], Kirsty Lu[1], Jessica Collins[1], Daniel G. Beasley[4], Ian B. Malone[1], David L. Thomas[5,6], Anna Barnes[7], Marcus Richards[3]†, Nick C. Fox[1]†, Jonathan M. Schott[1]†*

† Joint senior author

*Corresponding author

Professor Jonathan M. Schott

Dementia Research Centre

UCL Queen Square Institute of Neurology

Box 16, Queen Square, London WC1N 3BG

j.schott@ucl.ac.uk | + 44 (0) 20 3448 3553

1. Dementia Research Centre, Queen Square Institute of Neurology, University College London, London, UK
2. Lysholm Department of Neuroradiology, The National Hospital for Neurology and Neurosurgery, Queen Square, London
3. MRC Unit for Lifelong Health and Ageing at UCL, London, UK
4. School of Biomedical Engineering and Imaging Sciences, Kings College, London

For peer review only

5. Leonard Wolfson Experimental Neurology Centre, Queen Square Institute of Neurology, University College London, London, UK
6. Neuroradiological Academic Unit, Department of Brain Repair and Rehabilitation, Queen Square Institute of Neurology, University College London, London, UK
7. Institute of Nuclear Medicine, University College London Hospitals, London, UK

Word count: 4021 (excluding abstract, tables, figures and references)

For peer review only

ABSTRACT

Objective: To summarise the incidental findings detected on brain imaging and blood tests during the first wave of data collection for the Insight 46 study.

Design: Prospective observational sub-study of a birth cohort

Setting: Single-day assessment at a research centre in London, UK

Participants: 502 individuals were recruited from the MRC National Survey of Health and Development (NSHD), the 1946 British birth cohort, based on pre-specified eligibility criteria; mean age was 70.7 (SD: 0.7) and 49% were female.

Outcome measures: Data regarding the number and types of incidental findings were summarised as counts and percentages, and 95% confidence intervals were calculated.

Results: 93.8% of participants completed a brain scan (n=471); 4.5% of scanned participants had a pre-defined reportable abnormality on brain MRI (n=21); suspected vascular malformations and suspected intracranial mass lesions were present in 1.9% (n=9) and 1.5% (n=7) respectively; suspected cerebral aneurysms were the single most common vascular abnormality, affecting 1.1% of participants (n=5), and suspected meningiomas were the most common intracranial lesion, affecting 0.6% of participants (n=3); 34.6% of participants with complete blood result data had at least one abnormality on clinical blood tests (n=169), but few reached the pre-specified threshold for urgent action (n=11).

For peer review only

Conclusions: In older adults, aged 69-71 years, potentially serious MRI brain findings were detected in around 5% of participants, and clinical blood test abnormalities were present in around one third of participants. Knowledge of the expected prevalence of incidental findings in the general population at this age is useful in both research and clinical settings.

STRENGTHS AND LIMITATIONS

- A large number of participants underwent blood testing and brain imaging, at an almost identical age, and received feedback of incidental findings according to a pre-specified standardised protocol.

- Participants were recruited from the 1946 British birth cohort, a broadly representative sample of the population born in mainland Britain during one week in 1946.

- Participant perception regarding the disclosure of incidental findings was not formally assessed, nor was the impact on their longer-term health and psychological wellbeing.

For peer review only

INTRODUCTION

Incidental clinical findings are often discovered during the course of conducting research. An incidental finding can be defined as “a finding concerning an individual research participant that has potential health or reproductive importance…but is beyond the aims of the study.”[1] The primary aim of most research is to generate data and advance knowledge, rather than to diagnose health problems in participants, and there is currently no legal requirement for researchers in the UK to report incidental findings to participants.[2] There are, however, important ethical reasons for disclosing certain incidental findings to participants in appropriate circumstances, particularly when they relate to serious and potentially treatable conditions.[1] It is therefore important that studies have protocols in place for managing them. While there is no consensus on how this should be done, it is recommended that researchers weigh up the potential benefits and harm to participants of being informed, as well as considering the associated time and cost, both to the study and to publicly-funded health services.[2]

Incidental findings often lead to anxiety and have the potential to lead to unnecessary and invasive procedures for study participants.[3-5] Knowledge of the expected prevalence of incidental findings, based on clearly defined protocols for their determination, is important, allowing researchers to be better prepared for managing them, and enabling study participants to be appropriately informed as part of the consent process. Given the increasing use of neuroimaging in primary, secondary and tertiary care, such information is also useful in the clinical setting, where it can facilitate management decisions. For example, knowing the probability of detecting an abnormality unrelated to a patient’s symptoms might influence a clinician’s decision to recommend a brain scan in a patient presenting with a benign-sounding headache, or prompt discussion with the patient regarding the pros and cons of scanning.

For peer review only

The Medical Research Council (MRC) National Survey of Health and Development (NSHD) recruited 5362 individuals born in England, Scotland and Wales during the same week in 1946, and has followed them since birth, with over 2500 participants remaining in active follow up.[6] Insight 46 is a longitudinal neuroimaging sub-study of 502 MRC NSHD participants, which aims to investigate genetic and life course factors that contribute to healthy and pathological brain ageing, in particular cerebrovascular and Alzheimer's disease. It involves detailed clinical phenotyping, brain magnetic resonance imaging (MRI), cerebral ß-amyloid positron emission tomography (PET), and blood and urine collection, at two time points approximately two years apart. The full study protocol, which includes clear criteria for reporting incidental findings, has been described elsewhere.[7]

The aim of this study is to summarise the incidental findings detected on brain imaging and blood tests during the first wave of data collection for Insight 46. Several studies have reported rates of incidental findings in different samples previously,[8,9] but to our knowledge, none have reported on findings from a representative country-wide birth cohort.

METHODS

Recruitment

Individuals were recruited from NSHD participants who attended a study visit at age 60-64, who had previously indicated that they would be willing to consider participating in a study visit in London, and for whom relevant life course data were available (Supplementary File 1). NSHD participants who met these criteria were sent an information booklet about the study

For peer review only

and then recruited by a study doctor via telephone. Those with known contraindications to PET or MRI scanning were not recruited. Eligibility criteria were relaxed towards the end of the study, allowing inclusion of some individuals with a few missing life course data-points, in order to achieve the study's recruitment target.

Consent

The booklet sent to participants prior to their visit contained a detailed description of the study tests, including information about the study protocol with regard to incidental findings. Specifically, it stated that "we will inform you and your GP if any of the routine blood tests show any significant abnormalities" and we "will let you and your doctor know if there are any major abnormalities on the MRI scan (e.g. the presence of a tumour or a large aneurysm) which might affect your clinical care." It also emphasised that "being in a research study does not take the place of routine physical examinations or other appointments with your doctor and should not be relied upon to diagnose or treat medical problems." All participants provided written consent to participate (Supplementary File 2). Prior to collecting blood samples, the study doctor asked participants whether they wished to opt out of receiving a copy of their blood results. This option was given primarily to avoid overwhelming participants with feedback, with a view to contacting these participants only if they had actionable findings. They had to consent to their general practitioner (GP) being informed about them.

Neuroimaging

Participants underwent brain imaging on a single Biograph mMR 3 Tesla PET/MRI scanner (Siemens Healthcare). Participants were injected via an intravenous cannula with the ^{18}F

For peer review only

amyloid PET ligand Florbetapir at the start of the imaging session, and dynamic amyloid data was obtained over 60 minutes. MRI data were acquired simultaneously, including: volumetric T1-weighted, T2-weighted and fluid-attenuated inversion recovery (FLAIR) sequences; resting state functional MRI; multi-shell diffusion-weighted imaging; 3D gradient echo sequence for T2*-weighted/ susceptibility-weighted imaging; and arterial spin labelling (non-invasive perfusion imaging).

Blood tests

Participants provided blood samples for standard clinical tests including haemoglobin, platelet count, vitamin B12, urea, creatinine, random glucose and thyroid stimulating hormone (TSH). Samples were also taken for biomarker and genetic testing. Results of the clinical blood tests were reported back to the study team via email within 24 hours. Samples for biomarker and genetic testing were stored for future analysis.

Duty of care protocol for neuroimaging

Given that Insight 46 participants were scanned at a single centre with availability of consultant neuroradiologists, and due to the unique nature of the cohort, it was decided that MRI scans would have a radiologist review. All T1-weighted, T2-weighted and FLAIR MRI sequences were reviewed by one of two consultant neuroradiologists within two weeks of the scan. Other sequences were not routinely reviewed, on the basis that they do not form part of a standard diagnostic MRI examination in clinical practice. Neuroradiologists used a list of pre-specified reportable and non-reportable abnormalities to flag scans as being potentially reportable (Table 1). This list was adapted from the UK Biobank study, which classified findings as reportable

For peer review only

if they were potentially serious (i.e. life-threatening or likely to have a major impact on quality of life or function), based primarily on work performed by the German National Cohort.[5,10] Aneurysms <7mm were not considered reportable in keeping with the Rotterdam Scan study.[11] Neuroradiologists were also encouraged to flag scans with other unexpected findings if there was any possibility that further assessment might be required.

Reportable findings	Non-reportable findings
▪ Acute brain infarction<br>▪ Acute brain haemorrhage (note: not old bleeds)<br>▪ Intracranial mass lesions (note: not meningiomas in locations considered unlikely to cause problems)<br>▪ Suspected intracranial aneurysm or vascular malformation (including cavernomata) (note: not aneurysms <7mm in diameter)<br>▪ Colloid cyst of the 3rd ventricle<br>▪ Acute hydrocephalus<br>▪ Significant sinus disease with suspicion of underlying pathology (e.g. unilateral sinus opacification)<br>▪ Other unexpected, serious, or life-threatening findings	▪ White matter hyperintensities<br>▪ Suspected demyelination<br>▪ Non-acute brain infarction<br>▪ Chronic hydrocephalus<br>▪ Asymmetric ventricles<br>▪ Lipoma of the corpus callosum<br>▪ Developmental abnormalities<br>▪ Enlarged perivascular spaces<br>▪ Chiari malformation<br>▪ Hippocampal or other focal atrophy

Table 1. List of reportable and non-reportable MRI abnormalities (adapted from the UK Biobank, German National Cohort and Rotterdam Scan studies).[5,10,11]

The reporting process was performed electronically using the web-based data management tool XNAT (www.xnat.org), thereby providing an audit trail (Figure 1). Reporting radiologists downloaded images from the XNAT server, reviewed them, and then completed a radiological read report within XNAT (Supplementary File 3). This took around ten minutes per scan. Radiologists were not given any clinical information regarding participants, other than

knowing that they were all born in 1946. If a scan was flagged as potentially reportable, the study coordinator was automatically notified, and a multidisciplinary meeting was organised within four weeks of the study visit. The reporting neuroradiologist, study chief investigator, and other relevant members from the study team were present at this meeting. If the abnormality was agreed to meet criteria for being reportable, the team decided on a clinical action plan (e.g. further imaging and/or specialist referral). A study doctor then contacted the participant and their GP, by telephone and in writing, providing them with information about the MRI abnormality and the recommended clinical action. Since data were collected in an anonymised form, it was not possible to share the images for clinical use.

Results of the amyloid PET scan were not fed back to participants because of the diagnostic and prognostic uncertainties of using this test in cognitively normal individuals, and lack of disease-modifying treatments for people with amyloid pathology. These ethical considerations have been discussed elsewhere.[12]

Duty of care protocol for blood tests

Results of the clinical blood tests were reviewed by the study doctor and reported back to the participant's GP in writing within two weeks of the study visit. The participant was also sent a copy of these results if they had previously stated that they wished to receive one. If results fell outside the normal reference range (Table 2), these abnormalities were highlighted in a letter sent to both the participant and their GP, and participants were advised to discuss them with their GP. If results were deemed to be significantly abnormal, falling beyond pre-specified urgent action levels (Table 2), the study doctor contacted the participant and their GP by telephone within 48 hours of the study visit. These pre-specified levels were adapted from those

For peer review only

used at the NSHD whole cohort sweep at age 60-64.[6] They reflect values at which urgent action would be warranted in clinical practice and were developed in consultation with clinical scientists and physicians in the relevant field. Biomarker and genetic test results were not reported back to participants.

Blood test	Normal reference range	Urgent action level
Haemoglobin (male)	13.0-17.0 g/dl	<10 or >20 g/dl
Haemoglobin (female)	11.5-15.5 g/dl	<10 or >20 g/dl
Platelets	150-400 10^9/l	<100 or >1000 10^9/l
Vitamin B12	191-900 pg/ml	<100 pg/ml
Urea	1.7-8.3 mmol/l	>20 mmol/l
Creatinine (male)	66-112 µmol/l	>200 µmol/l
Creatinine (female)	49-92 µmol/l	>200 µmol/l
Glucose	3.5-10 mmol/l	>20 mmol/l
Thyroid stimulating hormone	0.27-5.5 mIU/l	<0.1 or >10 mIU/l

Table 2. Clinical blood tests, their normal reference ranges, and urgent action levels

Follow-up of incidental findings on brain MRI

While participants have not been systematically followed-up with regards to findings detected on brain MRI, data regarding outcomes has been obtained via different sources, mainly through telephone or written communication from participants, or through letters obtained from healthcare professionals.

Analysis

For peer review only

Data regarding the number and types of incidental findings, and the actions taken by the study team in response to them, were summarised as counts and percentages, and 95% confidence intervals (CIs) for proportions were calculated using the exact Clopper-Pearson method. Sex differences were assessed using a two-tailed two-sample test of proportions. A p-value <0.05 was considered significant. For brain MRI analyses, participants without a scan were excluded. For blood result analyses, participants were excluded if they had a missing value for the specific test or category being analysed. Very few participants had missing blood result values, primarily due to sampling or processing errors, and these were assumed to have occurred at random. All analyses were performed in STATA version 14.2. Estimated glomerular filtration rate (eGFR) was derived using the Modification of Diet in Renal Disease (MDRD) study equation: GFR (ml/min/1.73m^2) = 175 x (Scr/88.4)$^{-1.154}$ x (Age)$^{-0.203}$ (x 0.742 if female) where Scr equals serum creatinine in µmol/L.

Participant involvement

Study members helped in the design of the Insight 46 study through participation in focus groups. Participants were invited to complete evaluation forms following their study visit, outlining any positive or negative aspects of their experience. Results from the Insight 46 study will be disseminated to participants through newsletters and public engagement events.

RESULTS

502 participants attended a study visit in London from throughout mainland Britain between May 2015 and January 2018. Mean age was 70.7 (SD: 0.7) years and 49% were female. In

For peer review only

total, 181 participants had a reportable incidental finding on either brain MRI or clinical blood tests, and 45 participants had more than one reportable finding.

Brain MRI

93.8% of participants completed a brain scan (n=471). The most common reason for non-completion was claustrophobia (n=25). Other reasons included: being unable to lie comfortably in the scanner (n=3); concerns about radiation (n=1); possible metallic implants (n=1); and withdrawal from the study (n=1). 7.6% of scans (n=36) were flagged by neuroradiologists as having potentially reportable abnormalities for review. Following discussion between the reporting neuroradiologist and study chief investigator, 58.3% of these scans (n=21) were deemed to have an abnormality that fulfilled criteria for being reportable. Therefore, in total, 4.5% of all scans had an incidental finding that was reported to the participant and their GP. Details of flagged findings that were not deemed reportable are listed in Supplementary File 4.

Table 3 summarises the number and percentage of reportable MRI abnormalities by type and sex. The most common abnormalities were suspected vascular malformations and suspected intracranial mass lesions, which were detected in 1.9% (n=9) and 1.5% (n=7) of participants respectively. Suspected cerebral aneurysms were the most common vascular abnormality, affecting 1.1% of participants (n=5; Figure 2A). Suspected meningiomas were the most common intracranial lesion, affecting 0.6% of participants (n=3; Figure 2B). Females were more likely to have a reportable MRI abnormality than males (6.5% vs 2.5%; p=0.034).

With regards to management of incidental findings, further imaging was recommended in 66.6% of cases (n=14); specialist referral was advised in 57.1% of cases (n=12); advice

For peer review only

regarding medication and management was given in 19% of cases (n=4); and no action was recommended in 9.5% of cases where the abnormalities were found to be pre-existing and already being managed by the participant's local health services (n=2). Further information regarding follow-up and subsequent outcomes is summarised in Supplementary File 5.

For peer review only

	All (N = 471)		Male (N = 241)		Female (N = 230)	
	n	% (95% CI)	n	% (95% CI)	n	% (95% CI)
Any abnormality	21	4.5 (2.8, 6.7)	6	2.5 (0.9, 5.3)	15	6.5 (3.7, 10.5)
Acute brain infarction	-	-	-	-	-	-
Acute brain haemorrhage	-	-	-	-	-	-
Suspected intracranial mass lesion	7	1.5 (0.6, 3.0)	2	0.8 (0.1, 3.0)	5	2.2 (0.7, 5.0)
Suspected intracranial aneurysm or vascular malformation	9	1.9 (0.9, 3.6)	2	0.8 (0.1, 3.0)	7	3.0 (1.2, 6.2)
Colloid cyst of the 3rd ventricle	-	-	-	-	-	-
Acute hydrocephalus	-	-	-	-	-	-
Significant sinus pathology	3	0.6 (0.1, 1.9)	1	0.4 (0.0, 2.3)	2	0.9 (0.1, 3.1)
Other*	2	0.4 (0.0, 1.5)	1	0.4 (0.0, 2.3)	1	0.4 (0.0, 2.4)

Table 3. Number and percentage of reportable MRI abnormalities by type and sex

* possible keratocystic odontogenic tumour of right mandible (n=1); hyperintense area in the suprasellar cistern with differential diagnosis of small dermoid cyst, craniopharyngioma, or thrombosed anterior communicating artery aneurysm (n=1)

For peer review only

Standard clinical blood tests

Venepuncture was successful in over 99% of participants (n=498). Almost all participants chose to receive a copy of their clinical blood test results (n=496). There were missing blood result values in some participants (n=9) due to insufficient samples, lab errors, clumped platelets or a clotted sample. 34.6% of participants with complete blood result data had at least one abnormality on standard clinical blood tests (n=169). Of those participants with abnormalities, urgent action was required for 6.5% (n=11). In many of these cases (n=6), the participant's GP confirmed that the abnormality was pre-existing and already being managed. Table 4 summarises the number and percentage of blood test abnormalities by type and sex. Overall, males were significantly more likely to have at least one blood test abnormality than females (40.8% vs 28.0%; p=0.003). However, removing 'low creatinine' as an abnormality resulted in there being no significant difference between males and females (30.8% vs 26.4%; p=0.277).

For peer review only

	All (N = 498)		Male (N = 255)		Female (N = 243)	
	n/N	% (95% CI)	n/N	% (95% CI)	n/N	% (95% CI)
Any abnormality	169/489	34.6 (30.3, 39.0)	102/250	40.8 (34.6, 47.2)	67/239	28.0 (22.4, 34.2)
Polycythaemia	15/494	3.0 (1.7, 5.0)	11/254	4.3 (2.2, 7.6)	4/240	1.7 (0.5 4.2)
Anaemia	19/494	3.8 (2.3, 5.9)	14/254	5.5 (3.0, 9.1)	5/240	2.1 (0.7, 4.8)
Thrombocytosis	10/492	2.0 (1.0, 3.7)	2/252	0.8 (0.1, 2.8)	8/240	3.3 (1.4, 6.5)
Thrombocytopenia	11/492	2.2 (1.1, 4.0)	9/252	3.6 (1.6, 6.7)	2/240	0.8 (0.1, 3.0)
Elevated vitamin B12	10/495	2.0 (1.0, 3.7)	5/253	2.0 (0.6, 4.6)	5/242	2.1 (0.7, 4.8)
Low vitamin B12	16/495	3.2 (1.9, 5.2)	6/253	2.4 (0.9, 5.1)	10/242	4.1 (2.0, 7.5)
Elevated urea	40/497	8.0 (5.8, 10.8)	23/254	9.1 (5.8, 13.3)	17/243	7.0 (4.1, 11.0)
Elevated creatinine	17/497	3.4 (2.0, 5.4)	10/254	3.9 (1.9, 7.1)	7/243	2.9 (1.2, 5.8)
Low creatinine	41/497	8.2 (6.0, 11.0)	33/254	13.0 (9.1, 17.7)	8/243	3.3 (1.4, 6.4)
eGFR<60*	43/497	8.7 (6.3, 11.5)	15/254	5.9 (3.3, 9.6)	28/243	11.5 (7.8, 16.2)
Hyperglycaemia	21/497	4.2 (2.6, 6.4)	16/254	6.3 (3.6, 10.0)	5/243	2.1 (0.7, 4.7)
Hypoglycaemia	5/497	1.0 (0.3, 2.3)	1/254	0.4 (0.0, 2.2)	4/243	1.6 (0.5, 4.2)
Elevated TSH	13/496	2.6 (1.4, 4.4)	4/253	1.6 (0.4, 4.0)	9/243	3.7 (1.7, 6.9)
Low TSH	9/496	1.8 (0.8, 3.4)	-	-	9/243	3.7 (1.7, 6.9)
Urgent action	11/489	2.2 (1.1, 4.0)	3/250	1.2 (0.2, 3.5)	8/239	3.3 (1.5, 6.5)

Table 4. Number and percentage of clinical blood test abnormalities by type and sex

*eGFR (ml/min/1.73m^2) was calculated for this analysis to facilitate comparison with other studies; it was not reported back to participants

NB. Participants were excluded if they had a missing value for the specific test or category being analysed

For peer review only

DISCUSSION

In this study of older adults, aged 69-71, reportable incidental findings on brain MRI were present in 4.5% of scanned participants, with suspected vascular malformations and suspected intracranial mass lesions present in 1.9% and 1.5% of participants respectively. Clinical blood test abnormalities were common, affecting around one third of participants. However, very few blood test abnormalities required urgent action, and many of those that did were previously known to the participants' GPs and had already been acted upon.

Comparison with other studies

Due to the recent proliferation of neuroimaging research, incidental findings on brain MRI are often reported in the literature.[8,9] The reported prevalence varies between studies, likely reflecting differences in the definition of what constitutes an incidental finding, as well as variability in participant demographics and imaging protocols. Many imaging studies do not require routine review of all scans by a radiologist, and researchers will only ask for a radiologist opinion if an abnormality is identified incidentally by a radiographer during scanning or by researchers during data analysis.[13,14] Such studies may have lower detection rates, but are presumably less likely to publish data on incidental finding prevalence.

A 2018 systematic review reported an overall prevalence of 1.4% (95% CI 1.0% to 2.1%) for potentially serious brain incidental findings.[8] This is somewhat lower than the 4.5% (95% CI 2.8% to 6.7%) detected in Insight 46 participants, although this review consisted mainly of studies with younger participants using scanners of 1.5 Tesla or less. Most of the studies in this review used at least one radiological reader. Another systematic review reported a much higher

For peer review only

prevalence of 22% (95% CI 14% to 31%), likely due to their inclusion of all findings, regardless of their clinical seriousness.[9] Comparing specific abnormalities, namely suspected intracranial mass lesions and vascular malformations, in Insight 46 and 1936 Lothian Birth Cohort (LBC) revealed similar rates: 1.4% (95% CI 0.7% to 2.6%) and 2% (95% CI 1.1% to 3.3%) respectively in LBC subjects age 73, compared with 1.5% (95% CI 0.6% to 3.0%) and 1.9% (95% CI 0.9% to 3.6%) in Insight 46.[15] Results from another large population-based study, which included over 5800 subjects with a mean age 64.9 years, were marginally higher than Insight 46, with a prevalence of 2.5% (95% CI 2.1% to 2.9%) for suspected meningiomas and 2.3% (95% CI 2.0% to 2.7%) for suspected cerebral aneurysms.[11]

Most previous studies have found no significant difference in prevalence of potentially serious MRI brain findings by sex.[8] In Insight 46, however, higher rates were observed in female versus male participants. This was primarily driven by greater numbers of suspected intracranial mass lesions and vascular abnormalities in females, possibly due to the fact that meningiomas and cerebral aneurysms are more common in women than men.[11]

With regards to blood tests, Insight 46 tended to have either similar or lower rates of abnormalities than other studies. The prevalence of anaemia in a systematic review of studies involving community-dwelling older adults was 12%, which is somewhat higher than the 3.8% (95% CI 2.3% to 5.9%) detected in Insight 46 participants.[16] The prevalence of chronic kidney disease (CKD) stages 3-5 (eGFR <60ml/min/1.73m^2) is estimated to be around 6.1% in adults under 65 in England, rising to 13.5% for individuals aged 65-74, according to data collected in 2009-10 Health Survey for England and 2011 Census.[17] This is broadly in keeping with the rate of 8.7% (95% CI 6.3% to 11.5%) detected in Insight 46 participants. Vitamin B12 deficiency was detected in around 5% of individuals aged 65-74 years old in a

For peer review only

large UK-based study, compared with 3.2% (95% CI 1.9% to 5.2%) in Insight 46 participants.[18] Another large UK-based study found a prevalence of 7.9% (95% CI 6.4% to 9.6%) for elevated TSH and 6.0% (95% CI 4.7% to 7.4%) for low TSH in adults over 60 years old, somewhat higher than the 2.6% (95% CI 1.4% to 4.4%) and 1.8% (95% CI 0.8% to 3.4%) detected in Insight 46 participants.[19]

Discrepancies in the reported prevalence of blood test abnormalities between Insight 46 and other studies may be partly related to differences in laboratory assays, thresholds for defining abnormal values, and participant demographics. However, it also likely that certain blood test abnormalities are under-represented in Insight 46, since participants underwent clinical blood testing at a previous study visit aged 60-64 years old, and any abnormalities detected then were likely addressed at that time.[20] Indeed, comparing participant results at age 60-64 with those in the Insight 46 study revealed that: only 2 out of 9 participants still had anaemia; 8 out 27 still had an elevated TSH; and 5 out of 10 still had a low TSH.

Strength and weaknesses

A major strength of the Insight 46 study is that it involved a large number of participants who underwent brain imaging and blood testing, at an almost identical age, and received feedback regarding incidental findings according to a pre-specified standardised protocol. These participants were all recruited from the NSHD, the longest running British birth cohort, which has remained broadly representative of the population born in mainland Britain in 1946.[21] Distance from London was not found to be predictive of participation.[22]

For peer review only

High resolution MRI sequences were obtained using the same 3 Tesla PET/MR scanner for all participants, and images were systematically reviewed by one of two experienced consultant neuroradiologists. The process of reviewing scans was user-friendly and automated where possible, allowing scans to be reported within a short timeframe, thereby reducing the workload of the neuroradiologists. Scans were sometimes flagged for review, despite not having a reportable finding according to the study protocol, usually because the radiologist felt that the abnormality was serious enough to warrant further discussion. This was encouraged in order to avoid overlooking findings that might be considered actionable in the appropriate clinical context. In practice, however, this did not alter the number of findings reported to participants.

The duty of care protocol was developed in accordance with the MRC and Wellcome Trust framework on management of health-related research findings.[2] Any potentially serious brain MRI findings or blood test abnormalities were reported back to participants and their GPs, in keeping with the ethical principle of beneficence. Findings were not disclosed if tests lacked clinical utility or were not actionable, in order to minimise participant distress and harm. Participants were fully informed of the protocol for managing incidental findings as part of the consent process and were given the choice regarding whether they wanted to receive a copy of their blood results, thereby respecting their autonomy to make decisions about their own health. While it can be argued that research is generally not meant to benefit participants directly, many participants view medical input as an incentive to take part and there is an expectation that they will be informed of any serious findings. This needs to be balanced against the potential negative consequences of reporting incidental findings.[5]

A limitation of this study is that participant perception regarding the disclosure of incidental findings was not formally assessed, nor was the impact on their longer-term health and

For peer review only

psychological wellbeing. Many participants, however, gave informal feedback on post-visit evaluation forms that they appreciated being told about findings pertinent to their health and saw this as a benefit of being involved in the study. Moreover, almost all participants chose to receive a copy of their blood test results. These observations are consistent with results of a study commissioned by the Wellcome Trust and MRC, which found overwhelming public support for the disclosure of incidental findings in research, particularly in relation to serious and treatable conditions.[23] This is also supported by the work of several other studies.[3,5,24,25]

A further limitation of Insight 46 is that NSHD participants are all white Caucasian and, due to changing population demographics, results may not be directly generalisable to the current British population aged 70, or indeed younger populations. Furthermore, in separate analyses of recruitment to Insight 46, NSHD participants with higher educational attainment, non-manual socio-economic position and better self-rated health were more likely to take part.[22]

Implications and future work

The findings of this study will be relevant to future studies involving older adults, including clinical trials of secondary prevention drugs for Alzheimer's disease, which often involve MRI-based outcome measures and blood monitoring. Awareness of the expected prevalence of incidental findings on brain MRI and clinical blood tests in this age group, based on pre-defined protocols for their determination, should allow researchers to be better prepared for managing them, and participants to be better informed of their likelihood as part of the consent process.

For peer review only

The findings also have implications for clinical practice. In patients with benign-sounding headaches and normal neurological examination, for example, the chances of finding a serious intracranial cause on brain imaging is less than 1%.[26,27] Nonetheless, patients presenting with chronic headache frequently undergo brain imaging, usually to provide reassurance, and often at the patient's own request. These patients are rarely consented for the risk of discovering an incidental finding, despite the potential negative consequences. Greater awareness of the expected frequency and nature of incidental findings on brain imaging and blood tests should allow clinicians to counsel patients regarding their probability, and to balance this risk against the potential benefits of undergoing a test when deciding whether it is appropriate.

While the focus of this study was on potentially serious brain imaging findings, awareness of the prevalence of other incidental abnormalities, such as white matter disease, would also be useful from a clinical perspective. In separate analyses, the distribution of white matter disease burden in Insight 46 participants was found to be highly non-linear, making it difficult to define a threshold of abnormality.[28] Ongoing work investigating these changes, including longitudinal follow-up to assess their consequences, should help inform clinicians regarding their significance and management.

Further work is also needed to assess the implications of disclosing incidental findings in research studies, including the psychological effects and longer-term clinical consequences, as well as the impact on research integrity, particularly in longitudinal population studies where disclosure might lead to a biased sample. Outcome data regarding incidental MRI brain findings in Insight 46 were obtained, but this does not represent final diagnoses for all participants, nor does it include assessment of emotional impact. Due to the longitudinal nature of this study, it will be possible to collect these outcomes in a more systematic way after a

For peer review only

longer interval. This will be helpful to inform debates on the ethics of feeding back incidental findings to participants, adding to the work of several other ongoing studies.[3-5,11,24,25]

For peer review only

FIGURE LEGENDS

Figure 1. Simplified overview of the process for viewing and reporting scans using XNAT

Figure 2. (A) Sagittal T1-weighted image, demonstrating a 10mm aneurysm (arrow) arising from tip of the basilar artery. (B) Coronal FLAIR image, demonstrating a broad-based extra-axial lesion (asterisk) overlying the right superior frontal gyrus, consistent with a meningioma.

For peer review only

STATEMENTS

Funding

Insight 46 is funded by grants from Alzheimer's Research UK (ARUK-PG2014–1946, ARUK-PG2017-1946 PIs Schott, Fox, Richards), the Medical Research Council Dementias Platform UK (CSUB19166 PIs Schott, Fox, Richards), the Wolfson Foundation (PR/ylr/18575 PIs Fox, Schott), the Medical Research Council (MC_UU_12019/1 PI Kuh and MC_UU_12019/3 PI Richards), the Wellcome Trust (Clinical Research Fellowship 200,109/Z/15/Z Parker) and Brain Research Trust (UCC14191, PI Schott). AVID Radiopharmaceuticals (a wholly owned subsidiary of Eli Lilly) provide the PET amyloid tracer (Florbetapir) but had no part in the design of the study.

Competing interests

NCF's research group has received payment for consultancy or for conducting studies from Avid Radiopharmaceuticals, Biogen, Eisai, Elan, Eli Lilly Research Laboratories, GE Healthcare, IXICO, Janssen, Johnson & Johnson, Lundbeck, Pfizer, Roche, Sanofi-Aventis and Wyeth Pharmaceuticals. NCF receives no personal compensation for the activities mentioned above. JMS has received research funding from Avid Radiopharmaceuticals (a wholly owned subsidiary of Eli Lilly), has consulted for Roche Pharmaceuticals, Biogen, and Eli Lilly, given educational lectures sponsored by GE, Eli Lilly and Biogen, and serves on a Data Safety Monitoring Committee for Axon Neuroscience SE.

Ethics

Ethical approval was granted by the National Research Ethics Service (NRES) Committee London (REC reference 14/LO/1173, PI Schott).

Data Sharing

Data used in this publication are available to bona fide researchers upon request to the NSHD Data Sharing Committee via a standard application procedure. Further details can be found at http://www.nshd.mrc.ac.uk/data

Author Contributions

SEK and JMS conceived the manuscript. TDP, CAL, AK, SEK, SMB, HMS and AW recruited participants to the study. CH and SS reviewed and reported the MRI brain scans. TDP, CAL, AK, SEK, SMB, SNJ, KL and JC contributed to data collection. DMC, IBM, DLT and AB were responsible for setting up the imaging acquisition protocols, image processing and quality control. DGB was involved in data management. SEK analysed the data and drafted the initial manuscript. JMS, NCF and MR are Co-Principal Investigators of the study. All authors critically revised the manuscript and approved the submitted version.

Acknowledgements

We are very grateful to those study members who helped in the design of the study through focus groups, and to the participants both for their contributions to Insight 46 and for their commitment to research over the last seven decades. We are grateful to the radiographers and nuclear medicine physicians (Professor Ashley Groves, Dr Jamshed Bomanji, Dr Irfan Kayani) at the UCL Institute of Nuclear Medicine, and to the staff at the Leonard Wolfson Experimental Neurology Centre at UCL. We would like to acknowledge Dan Marcus and Rick Herrick for assistance with XNAT, Dr Philip Curran for assistance with data

For peer review only

sharing with the MRC Unit for Lifelong Health and Ageing, the DRC trials team for assistance with imaging QC, Mark White for his work on data connectivity, and Suzie Barker for her assistance with research governance.

REFERENCES

1. Wolf FM, Lawrenz FP, Nelson CA, et al. Managing incidental findings in human subjects research: analysis and recommendations. *J Law Med Ethics* 2008;36:219-211.
2. Medical Research Council, The Wellcome Trust. Framework on the feedback of health-related findings in research 2014. URL: https://www.mrc.ac.uk/documents/pdf/mrc-wellcome-trust-framework-on-the-feedback-of-health-related-findings-in-researchpdf/[date accessed – 25th January 2019].
3. de Boer AW, Drewes YM, de Mutsert R, et al. Incidental findings in research: a focus group study about the perspective of the research participant. *J Magn Reson Imaging* 2018;47:230-237.
4. Schmidt CO, Hegenscheid K, Erdmann P, et al. Psychosocial consequences and severity of disclosed incidental findings from whole-body MRI in a general population study. *Eur Radiol* 2013;23:1343-1351.
5. Gibson LM, Littlejohns TJ, Adamska L, et al. Impact of detecting potentially serious incidental findings during multi-modal imaging. *Wellcome Open Res* 2018;2:114

6. Kuh D, Pierce M, Adams J, et al. Cohort Profile: Updating the cohort profile for the MRC National Survey of Health and Development: a new clinic-based data collection for ageing research. *Int J Epidemiol* 2011;40:e1-e9.
7. Lane CA, Parker TP, Cash DM, et al. Study protocol: Insight 46 – a neuroscience sub-study of the MRC National Survey for Health and Development. *BMC Neurol* 2017;17:75.
8. Gibson LM, Paul L, Chappell F, et al. Potentially serious incidental findings on brain and body magnetic resonance imaging of apparently asymptomatic adults: systematic review and meta-analysis. *BMJ* 2018;363:k4577.
9. O'Sullivan JW, Muntinga T, Grigg S, et al. Prevalence and outcomes of incidental findings: umbrella review. *BMJ* 2018;361:k2387.
10. Bertheau RC, von Stackelberg O, Weckbach S, et al. Management of incidental findings in the German National Cohort. 2016 In: Weckback S, editor. Incidental radiological findings. First ed. Cham, Switzerland: Springer.
11. Bos D, Poels MM, Adams HH, et al. Prevalence, clinical management, and natural course of incidental findings on brain MR images: the population-based Rotterdam Scan Study. *Radiology* 2016;281:507-515
12. Harkins K, Sankar P, Sperling P, et al. Development of a process to disclose amyloid imaging results to cognitively normal older adult research participants. *Alzheimers Res Ther* 2015;7:26.
13. Booth TC, Waldman AD, Wardlaw JM, et al. Management of incidental findings during imaging research in "healthy" volunteers: current UK practice. *Br J Radiol* 2012;85:11-21.
14. Illes J, Kirschen M, Karetsky K, et al. Discovery and disclosure of incidental findings in neuroimaging research. *J Magn Reson Imaging* 2004;20:743-747

For peer review only

15. Sandeman EM, Hernandez Mdel C, Morris Z, et al. Incidental Findings on Brain MR Imaging in Older Community-Dwelling Subjects Are Common but Serious Medical Consequences Are Rare: A Cohort Study. *PLoS ONE* 2013;8:e71467.

16. Gaskell H, Derry S, Moore RA, et al. Prevalence of anaemia in older persons: systematic review. *BMC Geriatr* 2008;8:1.

17. Public Health England. Chronic kidney disease prevalence model. 2014. URL: https://assets.publishing.service.gov.uk/government/uploads/system/uploads/attachment_data/file/612303/ChronickidneydiseaseCKDprevalencemodelbriefing.pdf [date accessed - 25th January 2019].

18. Clarke R, Grimley Evans J, Schneede J, et al. Vitamin B12 deficiency and folate deficiency in later life. *Age Ageing* 2004;33:34-41.

19. Parle JV, Franklyn JA, Cross KW, et al. Prevalence and follow-up of abnormal thyrotrophin (TSH) concentrations in the elderly in the United Kingdom. *Clin Endocrinol* 1991;34:77-83.

20. Pierce MB, Silverwood RJ, Nitsch D, et al. Clinical disorders in a post war British cohort reaching retirement: evidence from the first national birth cohort. *PLoS ONE* 2012;7:e44857

21. Stafford M, Black S, Shah I, et al. Using a birth cohort to study ageing: representativeness and response rates in the National Survey of Health and Development. *Eur J Ageing* 2013;10:145-157

22. James SN, Lane CA, Parker TD, et al. Using a birth cohort to study brain health and preclinical dementia: recruitment and participation rates in Insight 46. *BMC Res Notes* 2018;11:885.

23. Opinion Leader, Wellcome Trust, Medical Research Council. Assessing public attitudes to health-related findings in research 2012. URL:

For peer review only

https://wellcome.ac.uk/sites/default/files/wtvm055196_0.pdf [date accessed - 25th January 2019].

24. Hegedus P, von Stackelberg O, Neumann C, et al. How to report incidental findings from population whole-body MRI: view of participants of the German National Cohort. *Eur Radiol* 2019 (ePub ahead of print).
25. Hegenscheid K, Seipel R, Schmidt CO, et al. Potentially relevant incidental findings on research whole-body MRI in the general adult population: frequencies and management. *Eur Radiol* 2018;23:816-826.
26. Sempere AP, Porta-Etessam J, Medrano V, et al. Neuroimaging in the evaluation of patients with non-acute headache. *Cephalgia* 2005;5:30-35.
27. Detsky ME, Mcdonald DR, Baerlocher MO, et al. Does the patient with headache have a migraine or need neuroimaging? *JAMA* 2006;296:1274-83.
28. Lane CA, Barnes J, Nicholas J, et al. Early midlife blood pressure influences late-life cerebrovascular disease and brain volumes but not beta-amyloid load – evidence from the 1946 British birth cohort. *Lancet Neurol* 2019 (in press)

For peer review only

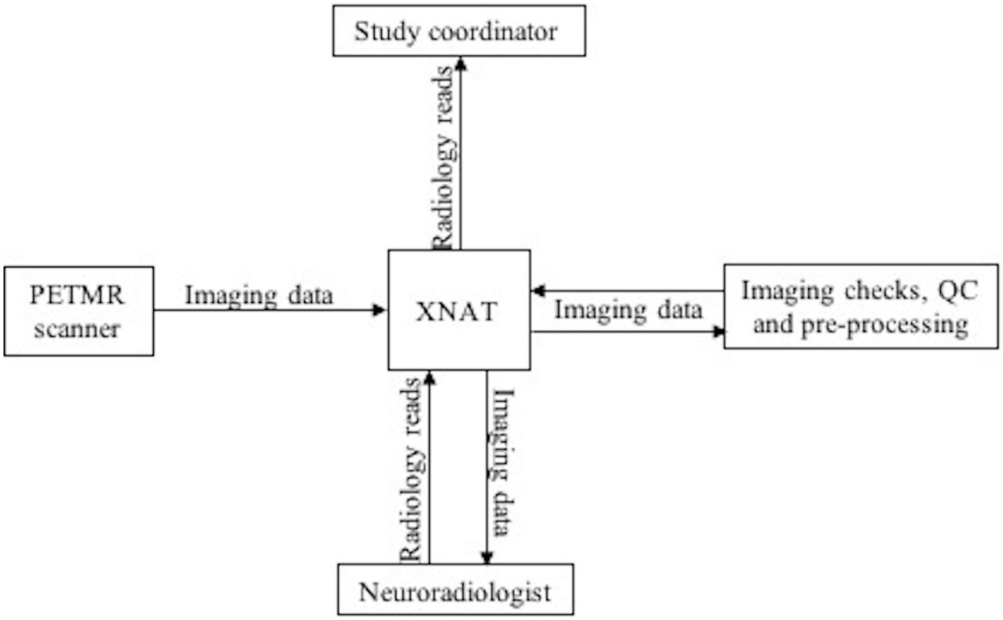

Figure 1. Simplified overview of the process for viewing and reporting scans using XNAT

147x91mm (300 x 300 DPI)

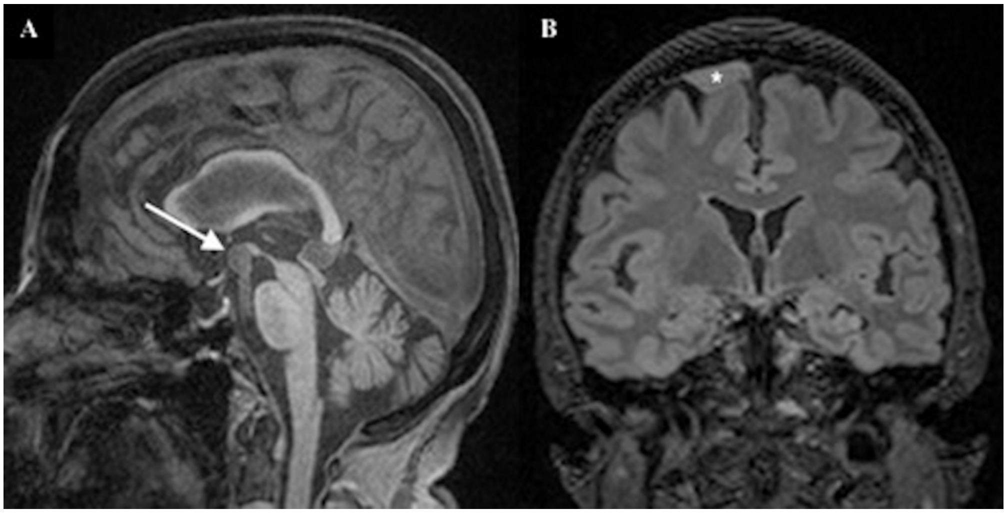

Figure 2. (A) Sagittal T1-weighted image, demonstrating a 10mm aneurysm (arrow) arising from tip of the basilar artery. (B) Coronal FLAIR image, demonstrating a broad-based extra-axial lesion (asterisk) overlying the right superior frontal gyrus, consistent with a meningioma.

160x81mm (300 x 300 DPI)

Supplementary File 1. Life course data required for Insight 46 eligibility*

1. Attendance at a clinic visit at age 60-64.
2. Parental socioeconomic position: at least one indicator of occupational social class or education.
3. Cognition: memory and processing speed from the 60-64 clinic visit AND at least one set of measures at ages 8, 11 or 15.
4. Early physical growth trajectories: birth weight and at least one measure of height and weight at ages 4-15
5. Educational attainment: highest qualification by age 26.
6. Mental health: teacher ratings of behaviour and temperament at ages 13 or 15, and at least one measure of affective symptoms at ages 36, 43, 53 or 60-64.
7. Blood pressure, lung function, adult height and weight: at least one measure of each at ages 36, 43, 53 or 60-64.
8. Health behaviours: at least one measure of smoking and physical exercise at ages 36, 43, 53 or 60-64.
9. Blood: either age 53 or 60-64 samples.

*Criteria were relaxed towards end of study to allow recruitment of 62 participants without a measure of lung function, smoking or physical exercise, in order to achieve recruitment target

For peer review only

MRC Unit for Lifelong Health and Ageing

UCL

MRC National Survey of Health and Development Neuroimaging sub-study
Participant Consent Form

Version 2.3:17-January-2015

PLEASE INITIAL BOXES – where you agree

1. I confirm that I have read and understand the information booklet for this follow-up of the MRC National Survey of Health and Development (version 2.3 dated 17 January 2015) I have had the opportunity to consider the information, ask questions about the study and have had these answered satisfactorily. ☐
2. I understand that certain assessments will be video recorded. ☐
3. I understand that relevant sections of any of my medical notes and data collected during the study may be looked at by responsible individuals from the MRC National Survey of Health and Development, from regulatory authorities or from the NHS Trust where it is relevant to my taking part in this research. I give permission for these individuals to have access to my records. ☐
4. I agree to have my research data electronically linked to other databases including those from primary care, Hospital Episode Statistics, NHS Health and Social Care Information Centre, research, clinical and administrative registries. To do this, I understand that my name, postcode, date of birth and NHS number will be shared with the NHS Health and Social Care Information Centre. ☐
5. I understand that the blood and urine samples will be used for research purposes only, and that no information found in the DNA will be given to me. ☐
6. I agree to the information and samples being used for health research carried out by the study team, and by scientists with research projects approved by the study's Data Sharing Committee. I understand that all analysis will be carried out and published using data only in numeric and anonymised form. ☐
7. I agree to the information and samples being held by UCL as the Data Controller. UCL is responsible for ensuring all data are securely stored, handled and used in accordance with the Data Protection Act. ☐
8. I agree that the results of the tests and measures listed in the GP letter be sent to my GP. ☐
9. I agree to take part in the above study. ☐

______________________	__________	______________________
Name of study member	Date	Signature
Name of person taking consent	Date	Signature

Thank you for agreeing to participate in the MRC NSHD Neuroimaging sub-study

When completed, 1 for study member, 1 for Institute of Neurology, and original for MRC NSHD

MRC Unit for Lifelong Health and Ageing at UCL 33 Bedford Place London WC1B 5JU
Telephone: 020 7670 5700 www.nshd.mrc.ac.uk

For peer review only - http://bmjopen.bmj.com/site/about/guidelines.xhtml

MRC National Survey of Health and Development Neuroimaging sub-study

PET/MRI Consent Form

Version 1.2:17-January-2015

PLEASE INITIAL BOXES – where you agree

1. I confirm that I have read and understood the participant information sheet (version 2.3 dated 17 January 2015) for the above study and have had the opportunity to ask questions. ☐
2. I understand that my participation is voluntary and that I am free to withdraw at any time, without giving any reason, without my medical care or legal right being affected. ☐
3. I agree to my GP being informed of my participation in the study and that my GP may be informed of any unexpected findings on the research scan. ☐
4. I understand that unexpected findings on the MR scan may be discussed with me. ☐
5. I agree to take part in the study. ☐

Name of study member	Date	Signature
Name of person taking consent	Date	Signature

Thank you for agreeing to participate in the MRC NSHD Neuroimaging sub-study

When completed, 1 for study member, 1 for Institute of Neurology, and original for MRC NSHD

For peer review only - http://bmjopen.bmj.com/site/about/guidelines.xhtml

Acute brain infarction	◯ No ◯ Yes
Acute brain haemorrhage	◯ No ◯ Yes
Intracranial mass lesion	◯ No ◯ Yes
Suspected intracranial aneurysm or vascular malformation (inc. cavernomata	◯ No ◯ Yes
Colloid cyst of the 3rd ventricle	◯ No ◯ Yes
Acute hydrocephalus	◯ No ◯ Yes
Significant sinus disease with suspicion of underlying pathology (e.g. unilateral sinus opacification	◯ No ◯ Yes
Other unexpected, serious, or life-threatening findings	◯ No ◯ Yes
Additional comments	
Flagged For Review	No

Save Revert Cancel

For peer review only

Supplementary File 4. Findings flagged by radiologists that were not considered reportable

- Moderately severe small vessel disease
- Extensive cortical and subcortical chronic changes, right cerebral lateral convexity – likely sequelae of chronic embolic infarcts in right M4 territories
- Radiological features (ventricular enlargement, sulcal crowding at the vertex, widening of the sylvian fissures) which could indicate normal pressure hydrocephalus in the appropriate clinical context
- Several T2-hyperintense lesions in the supratentorial white matter, including juxtacortical and periventricular foci, with a further lesion in the upper cervical spinal cord, suggestive of an inflammatory-demyelinating process
- Generalised frontoparietal volume loss with the impression of more marked midbrain atrophy, which in the appropriate clinical context raises the possibility of a neurodegenerative process
- Generalised neuroparenchymal volume loss, slightly more marked in the parietal lobes
- Altered FLAIR signal within some of the sulcal spaces of the left frontal lobe, which raises the possibility of previous subarachnoid blood or a leptomeningeal process
- Generalised involutional change (featuring areas of isolated sulcal widening) with more marked hippocampal volume loss
- T2-hyperintense intra-diploic lesion within the left frontal bone without associated cortical destruction or intracranial/extracalvarial extension, likely represents a haemangioma
- Moderately severe small vessel disease
- Relatively extensive patch of T2-hyperintense signal change in the cerebral white matter and pons, along with a cribiform appearance of the basal ganglia, in keeping with moderately severe small vessel disease
- Mild background small vessel disease, with a more confluent focus within the white matter deep to the right peri-rolandic region, which likely represents a focus of established subcortical ischaemia
- An ossified right temporal dural-based mass, likely meningioma
- Disproportionate neuroparenchymal volume loss affecting the left cerebral hemisphere, particularly the perisylvian region and temporal lobe, suspicious for neurodegeneration
- Moderate demyelination. Mild disproportionate cortical atrophy.

For peer review only

Supplementary File 5. Data regarding follow-up and subsequent outcomes of incidental findings on brain MRI

Incidental Finding	Outcome	Source
Possible aneurysm right ICA	Known about previously and already being followed up locally.	Participant
Probable right MCA bifurcation aneurysm	Seen by neurosurgery. Did not have further imaging or follow-up.	Participant
Possible right PCOMA aneurysm	Seen by neurosurgery. Had angiogram which showed 8mm right ICA aneurysm. Subsequently underwent endovascular treatment.	Clinician letter
Left cerebellar cavernoma with previous perinidal haemorrhage	No immediate action. Will have follow-up imaging at phase 2 visit.	Study documents
ACOMA aneurysm	Seen by neurosurgery. Had angiogram. To be repeated in 6 months to assess whether any interval change.	Participant
Possible cavernoma left mid cerebellar peduncle	Had contrast CT. Felt to be solitary cavernoma and deemed low risk.	Study documents
Multiple T2 hypointense lesions and possible pontine cavernoma	Given advice regarding BP control and avoidance of blood-thinners.	Study documents
Basilar tip artery aneurysm, partially thrombosed	Seen by neurosurgery. Underwent exploratory endovascular surgery, but no intervention performed. Subsequently died of stroke (aetiology unknown) around 17 months post-visit.	Participant/family
Prominent right thalamostriate vein with smaller adjacent connecting vessels	Seen by neurology. Further imaging confirmed developmental venous anomaly right frontal area and cavernomata. Given advice regarding BP control and avoidance of blood-thinners.	Clinician letter
Asymmetric configuration of pituitary fossa with T2-hyperintense signal on the left, and mild deviation of pituitary stalk to the right	Seen by neurosurgery. Further imaging confirmed small pituitary adenoma, which is being followed up with interval imaging.	Clinician letter
Signal change and mild swelling within medial aspect of right post-central gyrus	Previous breast cancer. Lung lesion on recent CXR. GP informed oncology re. brain scan abnormality, which in clinical context was felt likely to be a metastasis. Subsequently died.	GP/family
Small median mass at outlet of 4th ventricle without mass effect or hydrocephalus	Seen by neurology. Underwent contrast MRI brain and c-spine. Lesion thought to be subependyoma. Being followed up locally.	Clinician letter
Small well circumscribed extra-axial lesion centred on the pontine cistern on the left	Known meningioma. Already under follow-up. No action taken.	Participant

For peer review only - http://bmjopen.bmj.com/site/about/guidelines.xhtml

Right frontal parasagittal meningeal sessile mass	Seen by neurology. Contrast MRI confirmed meningioma and follow-up MRI at 1 year showed no interval change.	Clinician letter
Bulky pituitary gland with convex superior border	Seen by neurosurgery. Asymptomatic. Normal pituitary function tests. Baseline and repeat imaging at 1 year showed no interval change. No longer under follow-up.	Participant
Meningioma overlying left cerebellar hemisphere	Seen by neurology. Contrast MRI confirmed meningioma and follow-up MRI at 1 year showed no interval change.	Clinician letter
Right maxillary antrum almost completely filled by retained secretions.	Seen by ENT. As asymptomatic, not felt to require further tests.	Participant
Complete filling left maxillary sinus with expansion of the osteomeatal complex, raising possibility of an underlying obstructing lesion	Seen by ENT. Underwent nasoendoscopy – no underlying structural lesion. Advised nasal irrigation to decrease congestion. Discharged.	Participant
Complete opacification of the right maxillary sinus and adjacent nasal cavity	Seen by ENT. Had an MRI – no evidence of obstructing lesion. Prescribed antibiotics for sinusitis. Discharged.	Participant
Well circumscribed T1-hyperintense lesion within mandible on right, suggestive of a keratocystic odontogenic tumour	Seen by maxillofacial surgery. Tumour excised.	Participant
T1-hyperintense lesion within suprasellar cistern, differential of which includes dermoid, craniopharyngioma or ACOM aneurysm	Seen by neurology. Further imaging suggested lipoma or small dermoid cyst. No further action recommended.	Clinician letter

For peer review only

STROBE Statement—Checklist of items that should be included in reports of ***cross-sectional studies***

	Item No	Recommendation	Page No
**Title and abstract**	1	(*a*) Indicate the study's design with a commonly used term in the title or the abstract	1
		(*b*) Provide in the abstract an informative and balanced summary of what was done and what was found	3
**Introduction**			
Background/rationale	2	Explain the scientific background and rationale for the investigation being reported	5
Objectives	3	State specific objectives, including any prespecified hypotheses	6
**Methods**			
Study design	4	Present key elements of study design early in the paper	6
Setting	5	Describe the setting, locations, and relevant dates, including periods of recruitment, exposure, follow-up, and data collection	11
Participants	6	(*a*) Give the eligibility criteria, and the sources and methods of selection of participants	6,7
Variables	7	Clearly define all outcomes, exposures, predictors, potential confounders, and effect modifiers. Give diagnostic criteria, if applicable	8
Data sources/ measurement	8*	For each variable of interest, give sources of data and details of methods of assessment (measurement). Describe comparability of assessment methods if there is more than one group	7-10
Bias	9	Describe any efforts to address potential sources of bias	N/A
Study size	10	Explain how the study size was arrived at	6,7
Quantitative variables	11	Explain how quantitative variables were handled in the analyses. If applicable, describe which groupings were chosen and why	12
Statistical methods	12	(*a*) Describe all statistical methods, including those used to control for confounding	12
		(*b*) Describe any methods used to examine subgroups and interactions	12
		(*c*) Explain how missing data were addressed	12
		(*d*) If applicable, describe analytical methods taking account of sampling strategy	N/A
		(*e*) Describe any sensitivity analyses	N/A
**Results**			
Participants	13*	(a) Report numbers of individuals at each stage of study—eg numbers potentially eligible, examined for eligibility, confirmed eligible, included in the study, completing follow-up, and analysed	12,13
		(b) Give reasons for non-participation at each stage	11,13
		(c) Consider use of a flow diagram	-
Descriptive data	14*	(a) Give characteristics of study participants (eg demographic, clinical, social) and information on exposures and potential confounders	12
		(b) Indicate number of participants with missing data for each variable of interest	12,16
Outcome data	15*	Report numbers of outcome events or summary measures	13-17
Main results	16	(*a*) Give unadjusted estimates and, if applicable, confounder-adjusted estimates and their precision (eg, 95% confidence interval). Make clear which confounders were adjusted for and why they were included	13-17

For peer review only

		(*b*) Report category boundaries when continuous variables were categorized	N/A
		(*c*) If relevant, consider translating estimates of relative risk into absolute risk for a meaningful time period	N/A
Other analyses	17	Report other analyses done—eg analyses of subgroups and interactions, and sensitivity analyses	N/A
**Discussion**			
Key results	18	Summarise key results with reference to study objectives	18
Limitations	19	Discuss limitations of the study, taking into account sources of potential bias or imprecision. Discuss both direction and magnitude of any potential bias	21,22
Interpretation	20	Give a cautious overall interpretation of results considering objectives, limitations, multiplicity of analyses, results from similar studies, and other relevant evidence	18-20
Generalisability	21	Discuss the generalisability (external validity) of the study results	21,22
**Other information**			
Funding	22	Give the source of funding and the role of the funders for the present study and, if applicable, for the original study on which the present article is based	26

*Give information separately for exposed and unexposed groups.

Note: An Explanation and Elaboration article discusses each checklist item and gives methodological background and published examples of transparent reporting. The STROBE checklist is best used in conjunction with this article (freely available on the Web sites of PLoS Medicine at http://www.plosmedicine.org/, Annals of Internal Medicine at http://www.annals.org/, and Epidemiology at http://www.epidem.com/). Information on the STROBE Initiative is available at www.strobe-statement.org.

For peer review only
